# Genetic detection of peste des petits ruminants virus under field conditions: a step forward towards disease eradication

**DOI:** 10.1186/s12917-016-0940-0

**Published:** 2017-01-25

**Authors:** Waqas Ashraf, Hermann Unger, Sunaina Haris, Ameena Mobeen, Muhammad Farooq, Muhammad Asif, Qaiser Mahmood Khan

**Affiliations:** 1 National Institute for Biotechnology and Genetic Engineering (NIBGE), Faisalabad, Pakistan; 2 Pakistan Institute of Engineering and Applied Sciences (PIEAS), Islamabad, Pakistan; 3 Animal Production and Health Section, Joint FAO/IAEA Division of Nuclear Techniques in Food and Agriculture, Vienna, Austria

**Keywords:** Peste des petits ruminants virus (PPRV), Reverse-transcription loop mediated isothermal amplification (RT-LAMP), Nucleocapsid gene (N), Robust diagnosis

## Abstract

**Background:**

The devastating viral disease of small ruminants namely Peste des petits ruminants (PPR) declared as target for “Global Eradication” in 2015 by the Food and Agriculture Organization (FAO) and the World Organization for Animal Health (OIE). For a successful eradication campaign, molecular diagnostic tools are preferred for their specificity, efficacy and robustness to compliment prophylactic measures and surveillance methods. However, molecular tools have a few limitations including, costly equipment, multi-step template preparation protocols, target amplification and analysis that restrict their use to the sophisticated laboratory settings. As reverse transcription-loop mediated isothermal amplification assay (RT-LAMP) has such an intrinsic potential for point of care diagnosis, this study focused on the genetic detection of causative PPR virus (PPRV) in field conditions. It involves the use of a sample buffer that can precipitate out virus envelope and capsid proteins through ammonium sulphate precipitation and exposes viral RNA, present in the clinical sample, to the LAMP reaction mixture.

**Results:**

The test was evaluated using 11 PPRV cultures, and a total of 46 nasal swabs (*n* = 32 collected in the field outbreaks, *n* = 14 collected from experimentally inoculated animals). The RT-LAMP was compared with the reverse transcription-PCR (RT-PCR) and real-time quantitative RT-PCR (RT-qPCR) for its relative specificity, sensitivity and robustness. RT-LAMP detected PPRV in all PPRV cultures in or less than 30 min. Its detection limit was of 0.0001TCID_50_ (tissue culture infective dose-50) per ml with 10-fold higher sensitivity than that of RT-PCR. In 59.4% of the field samples, RT-LAMP detected PPRV within 35–55 min. The analytical sensitivity and specificity of the RT-LAMP were equivalent to that of the RT-qPCR. The time of detection of PPRV decreased by at least forty minutes or 3–4 h in case of in the RT-LAMP as compared with the RT-qPCR and the RT-PCR, respectively.

**Conclusions:**

The sensitive and specific RT-LAMP test developed in this study targeting a small fragment of the N gene of PPRV is a rapid, reliable and applicable molecular diagnostic test of choice under the field conditions. RT-LAMP requiring minimal training offers a very useful tool for PPR diagnosis especially during the “Global PPR Eradication Campaign”.

**Electronic supplementary material:**

The online version of this article (doi:10.1186/s12917-016-0940-0) contains supplementary material, which is available to authorized users.

## Background

Peste des petits ruminants (PPR) is an acute, highly contagious viral disease of small ruminants that is considered one of the major constraints for efficient small ruminant production in the developing countries. It is a notifiable, List-A disease to the OIE caused by peste des petits ruminants virus (PPRV) [1]. Since its emergence in West Africa in the 1940s, PPR has spread across vast regions of the Africa, Middle East, Arabian Peninsula, and Southern Asia [2]. PPR is endemic in Pakistan where approximately half (48.3–48.5%) of its small ruminant population is seropositive [3, 4]. The disease mainly exists in three pathogenic, per-acute, acute and sub-acute, forms. However, acute form is the most common form that causes 80–90% mortality in individual flocks [5]. Once the host is infected with PPRV, general viraemia develops within 4–6 days followed by high fever (104–106 °F), marked salivation, shallow erosions in the oral mucosa, serous to purulent oculonasal discharge, dyspnea, coughing, pneumonia and diarrhea [6, 7]. In the later stages, a sub-normal temperature of 101–102 °F and dehydration due to diarrhea can lead to hypovolumic shock and death of the affected animals [8].

PPRV; the causative agent of the disease, is a member of the genus *Morbillivirus*, in the family “*Paramyxoviridea*”. It is a negative-sense, single-stranded RNA virus. The genome of PPRV, viz. 3’-N-P-M-F-H-L-5’ encodes six structural proteins namely nucleocapsid, phosphoprotein, matrix, fusion, haemagglutinin and large polymerase [9–11]. Among these, nucleocapsid (N) protein gene is the most abundantly transcribed gene in the host cells and is therefore preferred target site for genomic detection of PPRV [12]. Conventionally, diagnosis of PPRV relied on serological techniques and virus isolation from clinical samples through its propagation in adaptable cell lines [13–15]. However, these techniques are labor-intensive and insensitive for PPRV detection especially during latent phase of the infection [16, 17]. In contrast, reverse transcription polymerase chain reaction (RT-PCR) is considered as standard diagnostic test for PPR worldwide [14, 18]. However, limitations associated with RT-PCR including its high cost and pre-requisite for scientific manpower render it unsuitable for low or middle-income country settings [19]. Accordingly, the World Health Organization (WHO) recommends that an ideal diagnostic test for such countries should meet the ASSURED (Affordable, sensitive, specific, user-friendly, robust, equipment free, deliverable to the end user) guidelines [20, 21]. The invention of isothermal technologies, like loop mediated isothermal amplification (LAMP) assay, for DNA amplification is a step-forward towards the development of ASSURED diagnostic tests [21]. LAMP based amplification of target nucleic acid is based on isothermal amplification of template DNA utilizing the strand displacement activity of Bst or Bsm DNA polymerase enzyme originated from *Bacillus stearothermophilus* or *Bacillus smithii*, respectively [22]. The stem-loop structures generated, at an initial phase, during LAMP initiate exponential amplification process that results in rapid accumulation of DNA amplicons of varying lengths [22, 23]. The LAMP products can simply be visualized with naked eye after the addition of fluorescent DNA-intercalating dyes such as SYBR Green I, propidium iodide and calcein to the reaction mixture. In addition, the generation of LAMP products can also be monitored on a real-time basis by measuring the change in fluorescence over a specified interval with an ESE-Quant tube scanner [23]. Here, we report the development of a one-step, single tube, N gene based RT-LAMP assay for direct detection of PPRV in clinical samples (swab extracts), cell culture supernatants, obviating the need for RNA extraction and cDNA synthesis step that can potentially make it a suitable diagnostic test for on-site detection of PPRV particularly during eradication campaign in low income country settings.

## Results

### Optimization of RT-LAMP Assay for the detection of PPRV in culture supernatants

**Fig. 1:**
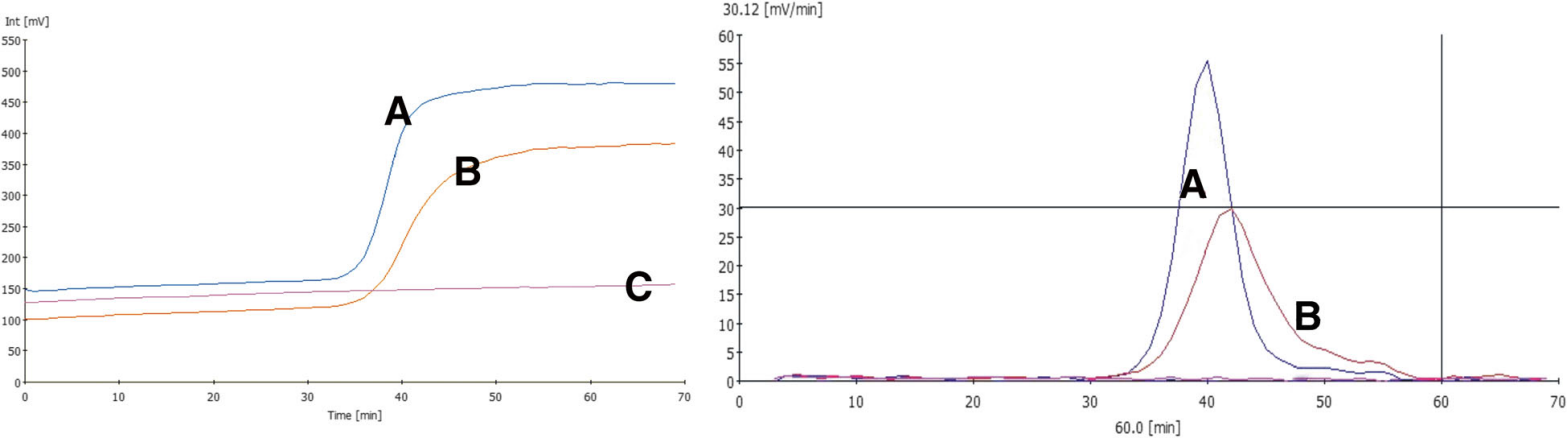
Optimization of primer concentration for N-gene based RT-LAMP assay. Curve A indicate amplification at optimized primers concentration while B indicate amplification efficiency at half of that primer concentration at which the increase in amplification signal per minute did not exceed threshold value of 30 mVolt/min. Negative control is indicated by a magenta colored line labeled as C

**Fig. 2:**
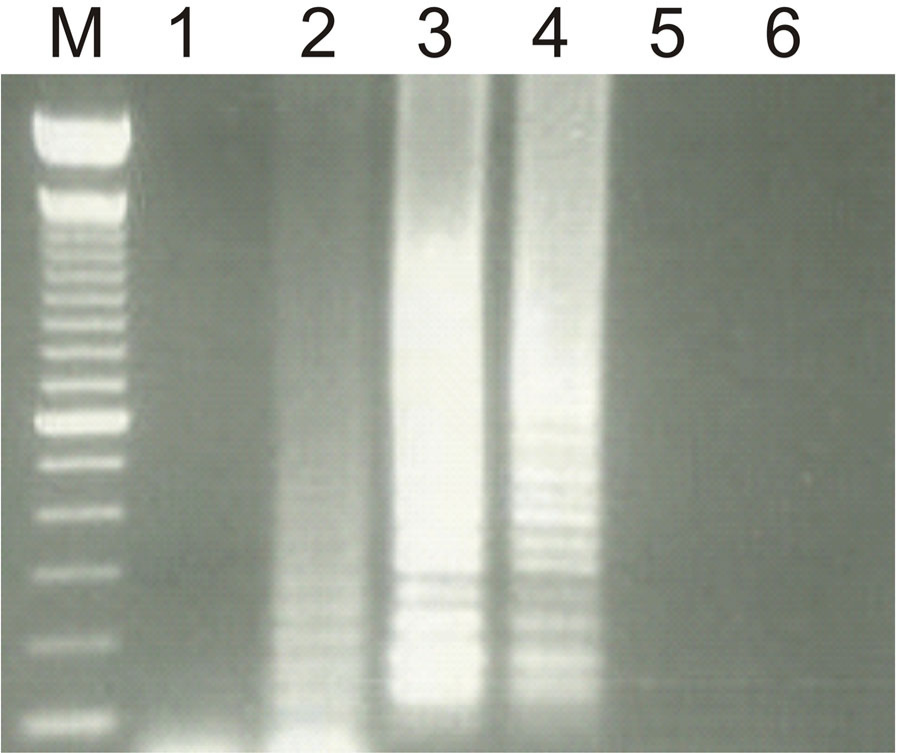
Gradient of incubation temperatures for N-gene based RT-LAMP assay. Lane 1: 50 °C; Lane 2: 53 °C; Lane 3: 55 °C; Lane 4: 58 °C; Lane 5: 60 °C; Lane 6: 62 °C. Precise amplification intensity was achieved at 58 °C

**Fig. 3:**
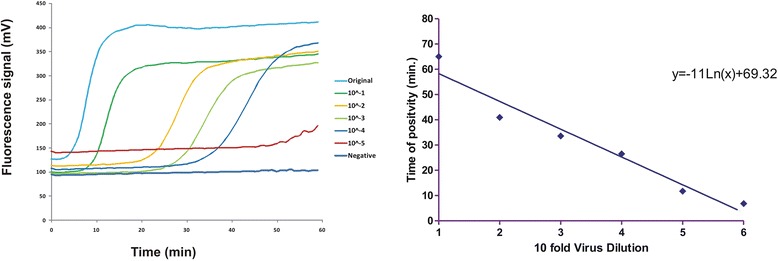
Sensitivity of N-gene based RT-LAMP assay. Fluorimetric curves in RT-LAMP assay were obtained from 10-fold Serial dilutions of fluid supernatant taken from cultured PPRV in CHS-20 cells harvested at appearance of 80% cytopathic effects. The test was able to detect dilution up to 10^−4^ within 60 min. Dilution 10^−5^ was detectable only after one hour

**Fig. 4:**
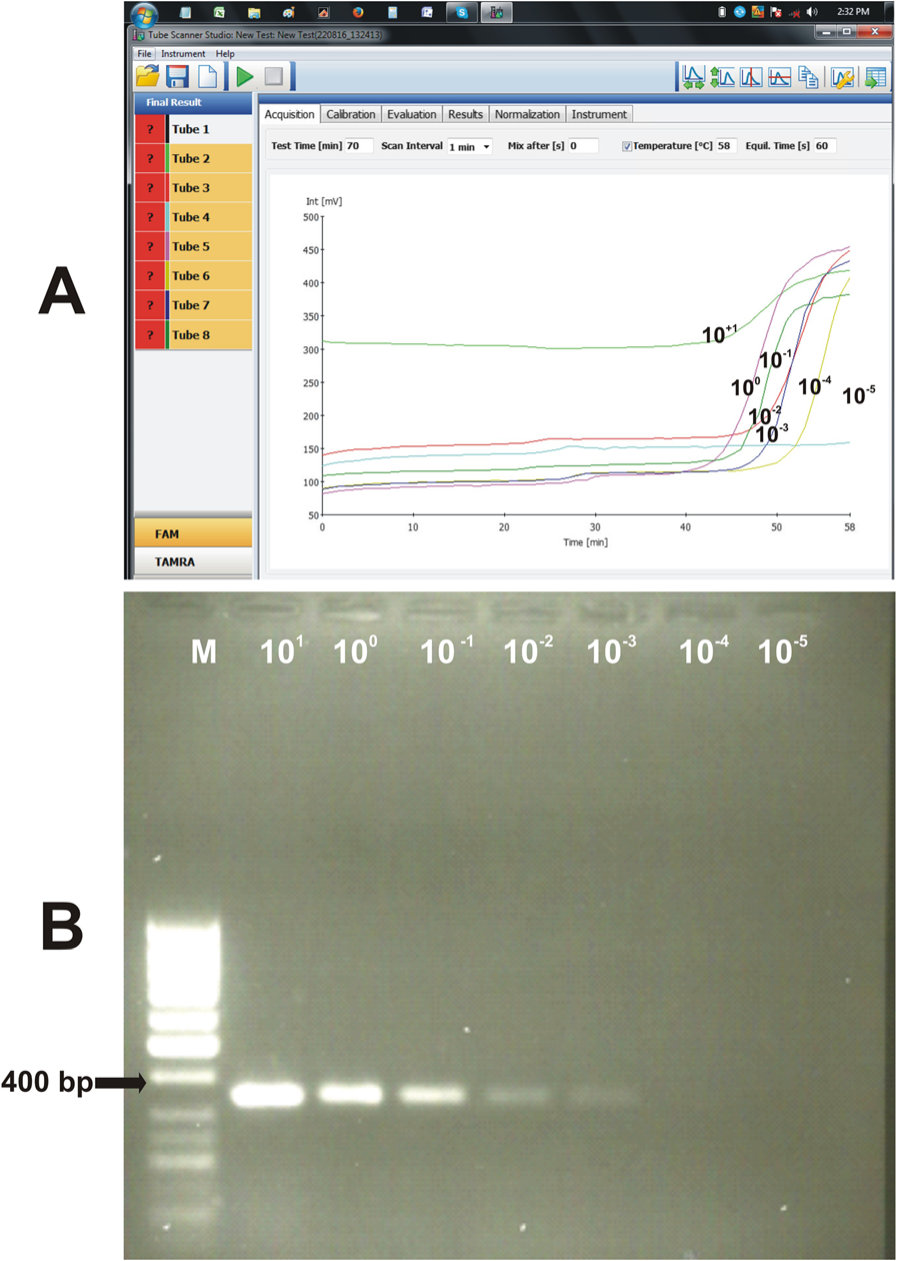
Sensitivity of N-gene based RT-LAMP assay in terms of TCID_50_/ml. Fluorimetric curves in RT-LAMP assay were obtained from 10-fold Serial dilutions of PPRV with starting concentration of 10TCID_50_/ml; Panel **a**. shows amplification curves generated from these dilutions within 60-min. Panel **b** shows the detection of these dilutions by RT-PCR recommended by OIE amplifying 351 bp of N gene [1, 14]. The RT-LAMP was able to detect up to 10^−4^TCID_50_/ml within 60 min as compared to 10^−3^TCID_50_/ml in case of RT-PCR

**Fig. 5:**
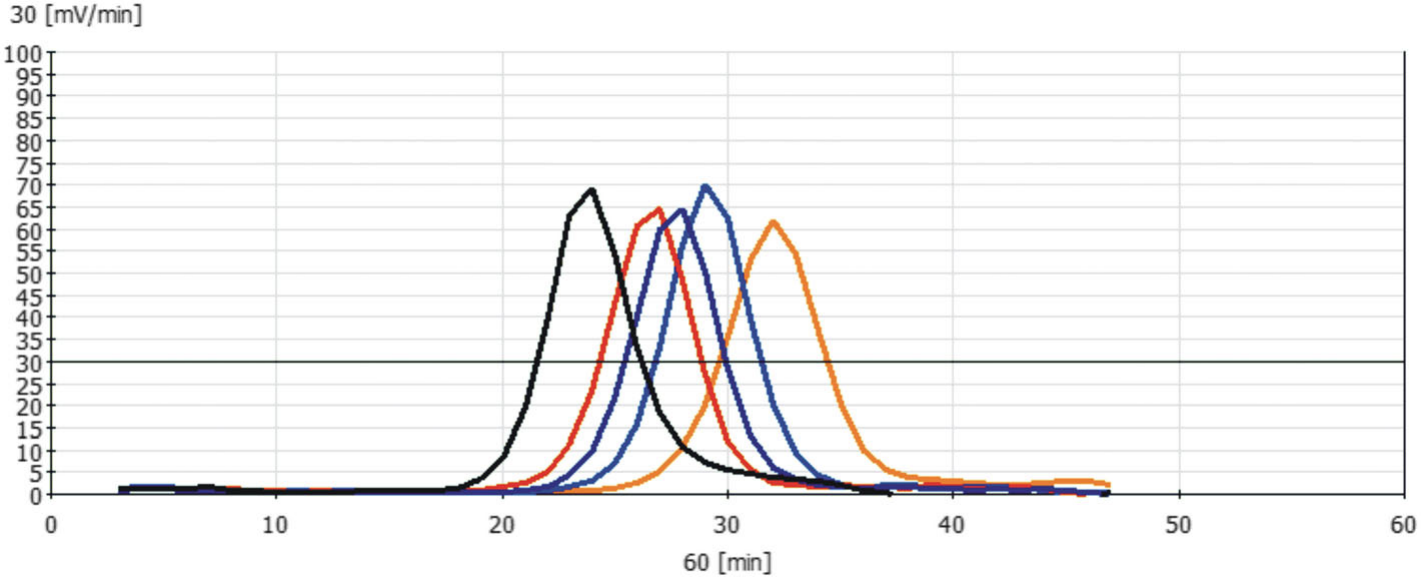
Threshold validation of positive reaction in RT-LAMP assay. The amplification curves shown in the figure were produced in the assay from culture supernatants of PPRVs. The curves were generated in response to change in fluorescence signal per minute during RT-LAMP assay by ESE Quant tube scanner software. According to threshold validation, amplification curves in positive reactions increased above threshold value of 30 mV per minute (indicated by horizontal line)

The success of an RT-LAMP assay mainly depends on three factors including primer concentration, optimal reaction temperature and template amount. Optimal primer concentration was found to be 0.25 μM for each of the outer primers (F3 and B3) and 1.25 μM for each of the inner primers (FIP and BIP) to achieve threshold increase of >30 mV for three consecutive readings. Once the primer concentration was reduced to half of the above-mentioned values, it compromised the efficiency of the reaction thus resulting in the drop in signal below the threshold value (Fig. 1). The optimal reaction temperature for each primer pair was determined using a gradient PCR (IQ5™, BioRad, USA) prior to its use in RT-LAMP assay. Accordingly, the assay was carried out at an increasing gradient of temperature that ranged from 50 °C to 62 °C. Gel electrophoresis of reaction products revealed amplification at three different temperatures including 53 °C, 55 °C and 58 °C. Among these, best amplification curve with high specificity was achieved at 58 °C (Fig. 2). Optimal template concentration for RT-LAMP assay was determined using a standard curve method. For this, tenfold serial dilutions of PPRV cell culture supernatant were prepared and subsequently subjected to RT-LAMP assay along with positive and no template controls (NTCs). The amplification curves for the positive control and the dilution factors viz; 10^−1^ to 10^−4^ showed typical four phases of amplification including the baseline, exponential phase, linear phase and a plateau within 60 min from the start of the assay. Amplification curve for 5^th^ dilution crossed threshold limit (30 mV/min) after 65 min (Fig. 3, Additional files 1 and 2) while NTC did not show any amplification. A standard curve was plotted with the log template concentration as the x value and the time of positivity (detection time, Td) as the y value. The y-intercept value (line representing the best fit) calculated using the least square method of linear regression was found to be 69.32 min. Accordingly, a maximum time limit of 60 min was set for the test to decide for positivity of the tested sample to ensure specific detection and rule out false positives. The detection limit of the assay (10^−4^ TCID_50_/ml) was 10 fold higher as compared with conventional RT-PCR (Fig. 4). Subsequent to the optimization of primer concentration, reaction temperature and template amount, culture soups of 11 PPRVs isolates, grown in CHS-20 cells, were detected by RT-LAMP. These cultures yielded amplification curves that climbed maximally up to 60-75 mV/min well above the 30 mV/min threshold during the LAMP reaction, indicating positive detection of PPRVs with a Td value of 30 or less than 30 min (Table 1, Fig. 5).

**Table 1:** PPRVs isolated from clinical samples by cell culture method from four district of Punjab Province of Pakistan and detected by RT-LAMP. Time points of detection (Ct values) of cultured PPRVs in RT-LAMP assay ranged from 12 to 30 min

No.	Type of sample	Origin	Place/year of outbreak	Cell culture (CHS-20 cells)	Td value (time point of first rise in fluorescence signal above threshold)
1	^a^NS	Sheep	DGK/2012	+	30
2	NS	goat	DGK/2012	+	12
3	NS	goat	MNW/2012	+	30
4	NS	goat	MNW/2012	+	10
5	NS	goat	MNW/2012	+	13
6	NS	goat	MNW/2012	+	14
7	NS	goat	SAH/2012	+	25
8	NS	goat	SAH/2012	+	20
9	NS	goat	SAH/2012	+	15
10	NS	goat	SAH/2012	+	12
11	NS	goat	CHK/2012	+	21

+, Each isolate name includes sample ID, acronym of the district of sampling and year, *DGK* DG Khan, *MNW* Mianwali, *CHK* Chakwal, *SAH* Sahiwal. *nd* not determined. ^a^ *NS* nasal swab

### Colorimetric and fluorimetric detection of RT-LAMP products

**Fig. 6:**
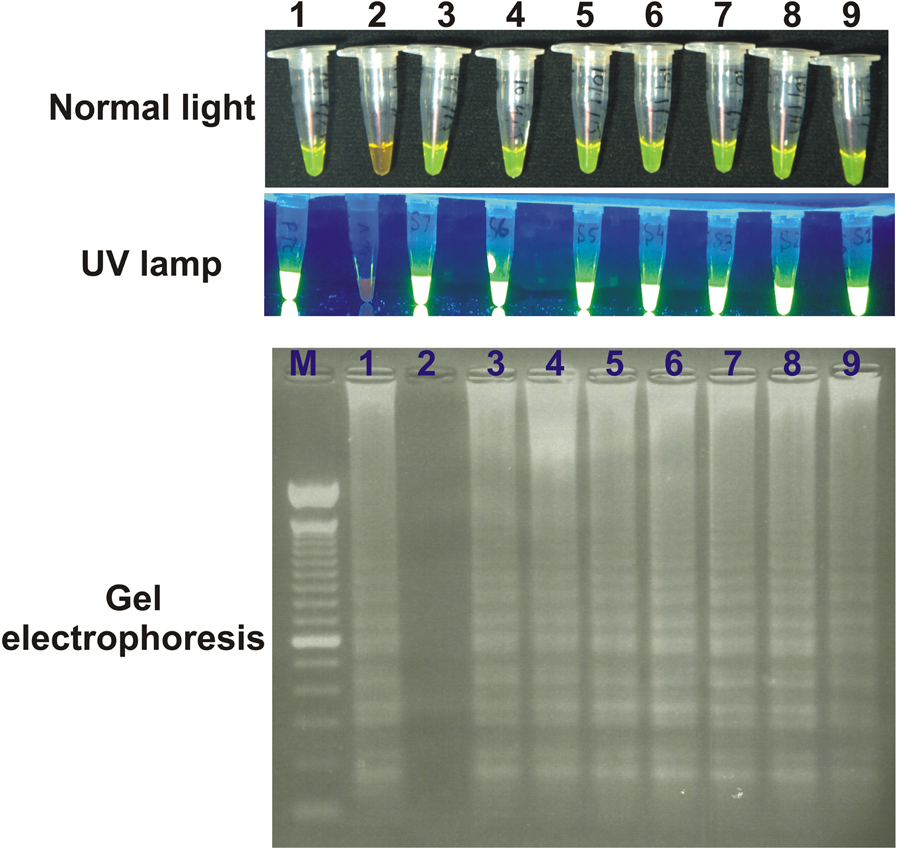
Visualization of RT-LAMP assay in normal light, Ultra Violet (UV) light and analysis by gel electrophoresis. Normal light: 1, positive reaction (green); 2, negative reaction (orange); UV light: 1, positive reaction (bright green fluorescent); 2, negative reaction (non-fluorescent). Gel electrophoresis: 1, positive reaction (ladder); 2, negative reaction (blank). 3 to 9 are positive clinical samples (same pattern as positive reaction)

SYBR green staining of RT-LAMP products resulted in a colour change, observable by naked eye, from orange to light green in case of positive reaction mixtures while the negative reaction mixture remains orange (Fig. 6). Upon UV light excitation, positive reaction products produced strong bright green fluorescence while negative control depicted slight background fluorescence (Fig. 6). These observations were in accordance with those of the gel electrophoresis as a ladder-like pattern of amplified DNA product was visualized in case of positive reactions while it was absent in case of negative control reactions (Fig. 6, Additional file 3).

### Robustness and specificity of RT-LAMP assay for detection of PPRV in experimentally inoculated animals

An equivalent of each swab extract collected, from experimentally inoculated goat and sheep, at 2, 4, 8, 10, 12, 14 day post-inoculation (dpi) was analyzed with RT-LAMP and RT-PCR to determine the relative robustness and sensitivity of RT-LAMP assay. During the course of experimental infection, RT-LAMP detected PPRV genome in the nasal secretions of goat from 2-dpi to 14-dpi (Table 2). It was, however, detected only on day-10 and 12 post-inoculation in sheep. The remaining samples did not produce amplification curves reaching beyond threshold limit of >30 mV increase in signal strength per minute and therefore, were considered as negative. In comparison to RT-LAMP, RT-PCR detected PPRV in the nasal discharges on 4-dpi to 14-dpi in the goat, whilst only on 10-dpi in sheep (Table 2, Additional file 4). Depending on the viral load, it was observed that RT-LAMP assay was able to detect the virus in the secretions of goat with Td value of 23 to 40 min which were equivalent to range of ct value of 31.5-36.4 as observed in RT-qPCR (Table 3). However, the pre-requisites of RNA extraction and cDNA synthesis in case of real-time RT-PCR made it take 76.5-80.3 min for the detection of these samples (Table 3, Additional files 5 and 6). RT-LAMP was found to be more robust than RT-qPCR by taking at least forty fewer minutes for detecting PPRV in these samples.

**Table 2:** Comparison of conventional RT-PCR and RT-LAMP in terms of detection of PPRV in nasal secretions

Species	Type of test	Days post inoculation							Total no. of positive samples
		2	4	6	8	10	12	14	
Goat	RT-PCR^a^	-	+	+	+	+	+	+	6
	RT-LAMP	+	+	+	+	+	+	+	7
Sheep	RT-PCR	-	-	-	-	+	-	-	1
	RT-LAMP	-	-	-	-	+	+	-	2

^a^These samples were also confirmed by RT-PCR mentioned in OIE terrestrial Manual [1]

**Table 3:** Comparison of robustness of RT-LAMP assay with real-time RT-PCR for detection of PPRV in nasal swab samples collected on alternative days from experimentally infected goat

Day post inoculation (dpi)	Detection time (Td) in RT-LAMP	Real-time RT-PCR	
		Ct value	Total time to reach Ct (minutes)^a^
2	39.5	36.4	80.33
4	23	31.5	76.5
6	39	34.5	78.7
8	37	34.7	78.9
12	40	34.2	78.5

^a^Total time includes RNA extraction + cDNA synthesis (50 min) + time to reach ct value in real-time PCR steps (50 s per cycle)

### Detection of PPRV in the animals affected in suspected outbreaks

**Fig. 7:**
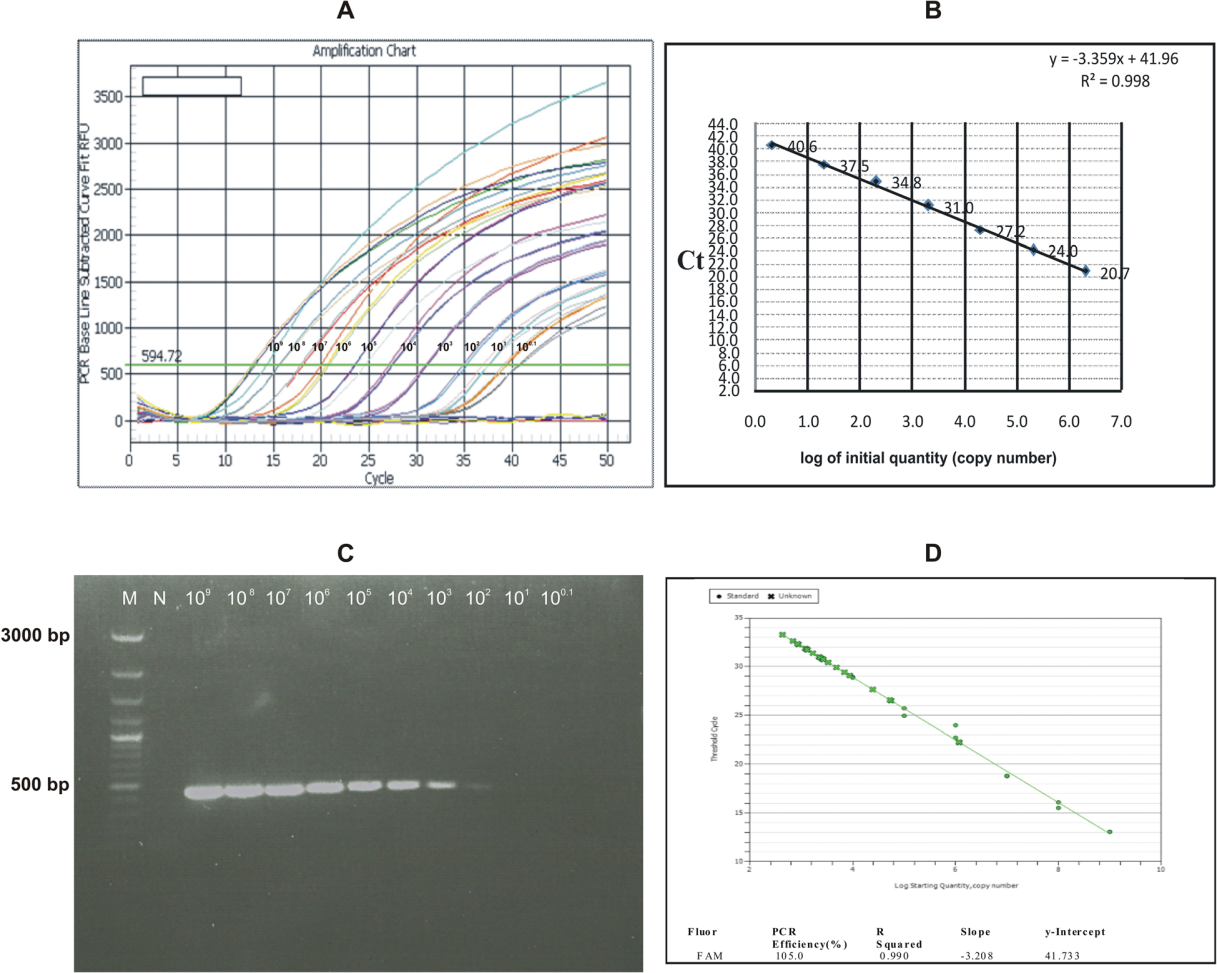
Limit of detection of RT-qPCR and its application for the detection of PPRV in clinical samples; Panel **a**. In the figure are shown amplification curves generated by serial dilution of cloned standard of PPRV N gene (463 bp fragment); Panel **b**. For the estimation of limit of detection, threshold cycle (Ct) values of standard dilutions from 2.06 × 10^6^ to 2.6 × 10^0^ were drawn against log values of copy number. Limit of detection was estimated to be ≈ 20 copies at 37.5 ± 0.9 cycle although Ct value of 40^th^ cycle was also detectable; Panel **c**. Detection limit of RT-PCR used in this study was up to 2.06 × 10^2^ copies of the plasmid; Panel **d**. The threshold cycle (Ct) values obtained by amplification of PPRV in clinical samples along with standard

A total of 32 nasal swabs collected from goats in field outbreaks at three districts of Punjab were subjected to the optimized RT-LAMP protocol along with positive and negative controls (Table 4). For threshold validation, only those samples were considered positive that produced amplification curve beyond 30 mV detection limit. Among the clinical samples, nineteen yielded an amplification curve with a Td value of 25-50 min (Table 4). However, thirteen clinical samples along with negative controls that consisted of measles and canine distemper viruses did not yield any amplification signal or a signal below threshold limit. In order to evaluate the sensitivity and robustness of RT-LAMP assay, equivalent set of the swabs was subjected to the conventional RT-PCR using N protein gene specific primers that generated a PCR fragment of ~450 bp in case of positive samples. Conventional RT-PCR detected PPRV in seventeen samples. These results indicated that RT-LAMP assay is somewhat as specific as the conventional RT-PCR assay; however, RT-LAMP detected PPRV in two more samples than RT-PCR. Comparatively, RT-LAMP was more robust in nature as it allowed quick detection of PPRV infection within 60 min as compared to 4–5 h in case of conventional RT-PCR that requires separate RNA extraction, cDNA synthesis, PCR and gel electrophoresis steps. RT-qPCR detected PPRV in *n* = 19 clinical samples by with Ct values of 22.2 to 33.5 (Fig. 7, Table 4, Additional files 7, 8, 9, 10, 11, 12 and 13). As all the samples that were positive in RT-qPCR were also positive in RT-LAMP, both tests were equally sensitive in the detection of PPRV.

**Table 4:** PPRV detection by RT-LAMP in clinical samples collected in outbreaks

District	Year of outbreak	Samples tested	RT-LAMP		No. positive in RT-PCR	No. positive in real-time RT-PCR (Ct value)
			Positive	Negative		
Mianwali	2012	08	04	04	04	04 (22.3–26.5)
Chakwal	2012	04	03	01	03	03 (31.7–33.1)
Sahiwal	2014	20	12	08	10	12 (26.2–33.3)
Total (percentage)	-	32	19 (59.4%)	13 (40.6%)	17 (59.4%)	19 (59.4%)

## Discussion

The overall aim of this research was to develop a reliable and cost-effective diagnostic test for on-site detection of peste des petits ruminants virus (PPRV) infection in sheep and goats based on LAMP technology that might serve as a foundation for the development of point of care diagnostic tests for different infectious diseases of livestock prevalent in Pakistan. We selected PPR disease to start with on the basis of two reasons viz. i) economic significance, ii) close relevance to its prototype, Riderpest (cattle plague) that has been effectively eradicated from the face of the earth. Current diagnostic tests for PPR involve the use of immunoassays to detect the antibodies against PPRV, virus isolation using adaptable cell lines and PPRV genome detection using RT-PCR or Real Time RT-PCR [13–18]. However, these techniques require costly equipment and highly trained manpower that render these tests unsuitable for on-site application.

**Fig. 8:**
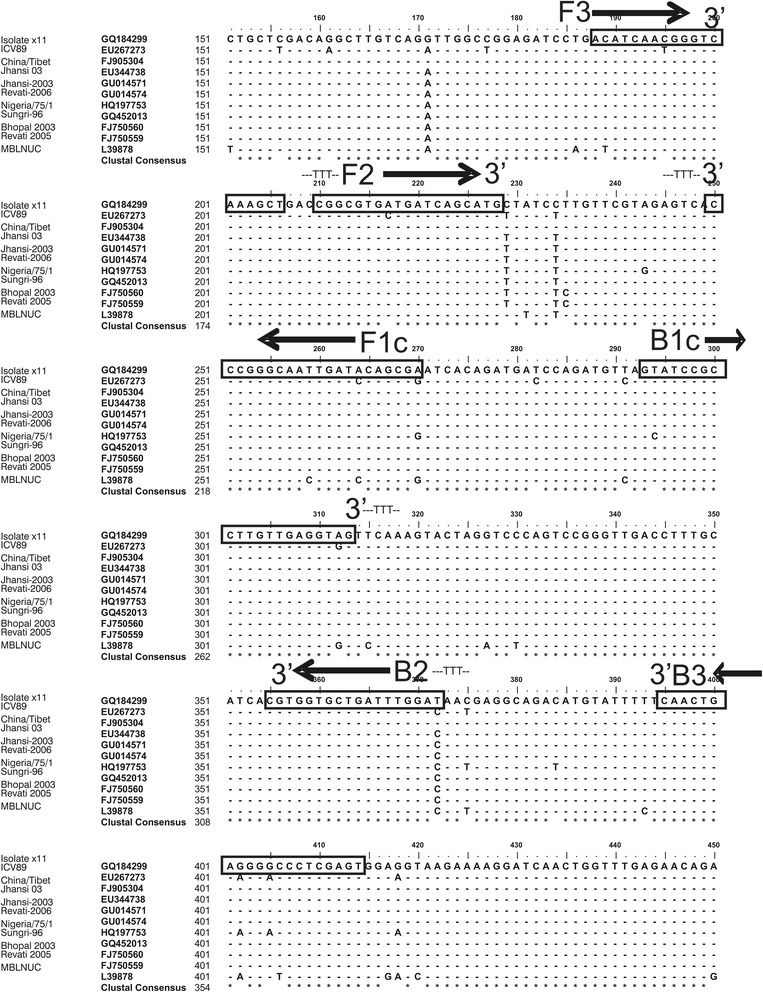
Locations of RT-LAMP primers along the nucleoprotein gene sequence of PPRV. Primers are indicated by solid boxes and solid line arrows in the right (→) and left orientation (←) indicate forward (F3, F2 and B1c) and reverse primers (F1c, B2 and B3), respectively. The length of product was 187 as limited by Forward (F3) and backward outer (B3) primers

Recently, LAMP based diagnostic tests have been developed for various viral diseases (PPR, Avian Influenza, FMDV) [24–26]. Although, these tests are relatively inexpensive and robust in nature yet challenges associated with LAMP technology including template preparation protocols (RNA extraction and cDNA synthesis) need consideration to take it to the point of care diagnostics. Our protocol involves the use of a sample buffer that can precipitate out virus envelope and capsid proteins through ammonium sulphate precipitation and exposes viral RNA, present in the clinical sample, for its subsequent amplification through LAMP reaction. Hitherto, N gene mRNAs are preferred target for molecular detection of PPRV because it is the most abundantly transcribed viral gene in all morbilliviruses [14, 27]. Hence, in this test, highly conserved region at the 5′ end of nucleocapsid gene specific to PPRV was targeted for primer designing to ensure specificity and sensitivity (Fig. 8). For an unknown reason, we found this protocol more effective for PPRV detection in fresh samples (cell culture supernatants, swab extracts) as compared to previously stored samples that had undergone many freeze-thaw cycles.

The monitoring system of ESE Quant tube scanner provided the ease of spatio-temporal curve analysis during the RT-LAMP reaction. Accordingly, the test can be used even in worse field conditions where electricity is not available and transportation facilities are inadequate. To ensure specificity while maintaining high sensitivity, an algorithm is incorporated into the tube scanner software that allows threshold validation of the amplification curve to consider it as positive only if the increase of signal (slope) above the mean of the baseline is > 30 mV for at least 3 consecutive measurements. This method of curve validation adds to the confidence of RT-LAMP result and, therefore, is more reliable than gel-electrophoresis and colorimetric analysis. By doing so, all seven samples of experimentally infected goat and only two out of total of seven samples of sheep, collected on alternative days for a period of two weeks, were considered as positive. Because all the samples that were positive by RT-PCR was also positive in RT-LAMP, RT-LAMP was rather as specific as conventional RT-PCR. Nonetheless, RT-LAMP detected PPRV in these samples 4–5 h earlier than RT-PCR. Comparative sensitivity (100%) of the RT-LAMP with RT-qPCR was assured by the fact that both detected nineteen clinical samples, collected in field outbreaks; while conventional RT-PCR (89.5% sensitive) could not detect PPRV in two samples that were positive both in RT-LAMP and RT-qPCR. The specific detection of RT-LAMP can be attributed to its intrinsic stringency by single small fragment (≈200–250 bp) simultaneously by utilizing six primers, while the large amount of DNA produced during the reaction ensures high sensitivity [22–26]. LAMP can multiply the target DNA 10^9^ to 10^10^ times the starting amount and detect 0.01 to 10 plaque-forming units (pfu) of viral particles [28]. The sensitivity of the RT-LAMP developed in this study is comparable to previously reported LAMP assays targeting either M or N gene for the detection of PPRV [24, 29]. In our study, RT-LAMP assay was10-fold more sensitive (detection limit of 0.0001TCID_50_/ml) and 3–4 times more robust than conventional-RT-PCR with a detection limit of 0.001TCID_50_/ml as reported by Couacy-Hymann et al. [14]. Previous protocols of LAMP, however, have used extracted RNA for subsequent synthesis of cDNA prior to its amplification by LAMP. The essential steps of RNA extraction and its reverse transcription require a sophisticated laboratory environment and increase the time of detection. The leading benefit of the RT-LAMP developed in this study for the detection of genome of PPRV directly by using the virus culture supernatant or clinical samples as such without a need for separate RNA extraction and/or cDNA synthesis ensure its application in the field. Due to the added benefits of higher sensitivity, robustness and simplicity, N gene based RT-LAMP can be a proficient alternative to the conventional RT-PCR especially in open field conditions where cold chain of sample transportation and highly equipped laboratories are not available.

Under UV light, a bright green fluorescence is observed in case of positive reaction (on addition of SYBR-green). This ability of the RT-LAMP assay to differentiate between positive and negative samples can be very useful especially in the field conditions even if the tube scanner is not available. Due to the high sensitivity of the test, it can be assumed that if a test is negative by threshold validation, colorimetric analysis and gel-electrophoresis, it would be essentially negative [21–26]. For that reason, RT-LAMP can be used for the detection of PPRV antigen in eradication campaigns to confirm the absence of disease.

The world population is experiencing continuous growth at an annual rate of 1.1% and estimated to reach 9.1 billion by 2050 [30]. Keeping it in the view, The Food and Agriculture Organization (FAO) of the United Nations estimates that the overall food production will need to increase by 70% in order to feed this projected world population thereby increasing pressure on the livestock sector to meet the growing demand for high value animal protein. Along with developed countries, where maximum livestock potential is already being utilized, developing countries like Pakistan, which have a lot of unexplored potential for livestock, need to contribute towards global food security. In these countries, there are certain challenges faced by livestock sector to cope up with these needs, among those, infectious diseases remain a key constraint and must be controlled for efficient livestock production. Accordingly, successful disease control or eradication program should be launched that would mainly rely on the prophylactic measures, surveillance & monitoring methods as well as the efficacy & robustness of the diagnostic tests. LAMP based diagnostic methods have taken the lead over other costly ones for being robust, economic and sensitive. RT-LAMP, developed in this study, for robust, specific on-site diagnosis of PPRV nucleic acid under field conditions is very practicable in the disease diagnosis during eradication of PPR. It offers a unique utility in the “Global PPR Eradication Campaign” launched recently, in the year 2015, by the FAO and OIE [31].

## Conclusions

These laboratory, experimental and field evaluation of the LAMP method developed in this study for the on-site diagnosis of PPR showed that it is sensitive, robust, and easy diagnostic method and can prove to be very useful to the field practitioners. These features make it an inimitably advantageous nucleic acid detection system for the confirmation of PPRV infection under the field conditions. It is a sensitive as well as stringent assay of choice for quick detection of the virus in epidemics or during regular abattoir inspections or screening programs targeting PPRV eradication.

## Methods

### Cell lines, viruses and clinical samples

Vaccine strain of PPRV, Nigeria 75/1 was used as a reference strain in this study. In addition to 11 cultured PPRV strains a total of 32 nasal swabs collected from goats in three suspected PPR outbreaks during 2012–2014 were evaluated for virus detection. Those suspected goats were owned by herdsmen who were well informed about the purpose of sample collection and their consent was taken prior to collection of swab samples. The virus isolation from individual samples was carried out using CHS-20 cell line (kindly provided by Dr. Adama Diallo, IAEA Laboratories, Austria) that stably express signaling lymphocyte activation molecule (SLAM), a cell surface receptor for PPRV [8].

### PPRV inoculation of sheep and goat

Two animals, one goat and one sheep bought from local livestock market, at the age of 5–6 months were tested negative for the presence of antibodies against PPRV using competitive enzyme linked immunosorbent assay (cELISA) [32] and acclimatized to controlled environment for two weeks. These were also confirmed as negative for PPRV antigen by RT-PCR [33] prior to their inoculation intravenously with 1 ml of 10^2^ (TCID_50_) PPRV strain 17BLK, which was isolated from a clinically infected goat in 2012 [34]. These animals were bought from local market and kept under controlled conditions at NIBGE animal house and were examined on a daily basis for the appearance of any clinical signs and symptoms up to 14 days post infection (dpi). Experimental samples including blood and nasal swabs were collected from each animal on alternate days up to 14dpi. The samples were stored at −80 °C until further use.

### Primer designing

Full-length nucleotide sequences of N protein gene of lineage-IV PPRVs along with vaccine strain (Nigeria/75/1) were retrieved from the GenBank database and aligned using MegAlign 5.00, DNA star Inc., software to identify conserved regions. Analysis of the prospective target regions was performed with the LAMP primer design software (www.http://primerexplorer.jp/elamp4.0.0/) for the automated selection of the primers. A set of primers was finally selected that included an outer pair consisting of forward outer (F3) and backward outer (B3) primer, an inner pair consisting of forward inner (FIP = FIc + F2) and backward inner (BIP = B1 + B2c) primer as mentioned in Table 5. The location of these primers on the conserved target region is shown in Fig. 8.

**Table 5:** N gene based primers set used in detection of PPRV by RT-LAMP assay

Primer ID	Type	Sequence (5ˊ to 3ˊ)	Position*	Length (bases)	Predicted length of amplified segment
PPRV F3	Forward outer	ACATCAACGGGTCAAAGCT	295–313	19	187 bp
PPRV B3	Backward outer	ACTCGAGGGTCCTTCAGTTG	521–502	20	
PPRV/FIP	Forward inner (F1c + F2)	CCGCTGTATCAATTGCCCGGGTTTTCGGCGTGATGATCAGCATG	376–357/317–335	44	
PPRV/BIP	Backward inner (B1 + B2c)	GCATCCGCCTTGTTGAGGTAGTTTTTTTGTCCAAATCAGCACCACG	400–421/481–462	46	

*The position of primers is indicated as per sequence position of complete genome of PPRV (Accession #hq197753)

### Sample preparation and RT-LAMP reaction

Two microliter (2 μl) of swab extract or cell-culture supernatant was added to 30 μl of RT-LAMP buffer (66 mM Tris-HCl pH8.8, 32 mM KCl, 32 mM (NH4)_2_SO_4_, 16 mM MgCl_2_, 0.3% (v/v) Tween 20) and incubated at room temperature for five minutes. Fifteen microliter (15 μl) of this sample mixture was added to 10 μl of reaction mixture providing final concentrations of 0.4 mM dNTP, 460 mM trehalose and 0.4X EvaGreen® dye. A ready to use primer mix was added providing a final concentration of 0.25 μM of each of the outer primers (F3 and B3) and 1.25 μM of each of the inner primers (FIP and BIP). Amplification of the target genome segment of PPRV based on strand displacement activity was achieved using 8 units per reaction of Bsm polymerase (FermentasThermo Scientific, St. Leon-Rot, Germany). The assay was carried out at 58 °C in an ESE-Quant Tube Scanner TS95 (Qiagen, Hilden, Germany) and the increase in fluorescence signal was recorded once per minute for 60 min.

### Fluorometric and gel electrophoresis based analysis of RT-LAMP products

ESE-Quant Tube Scanner was set at 6- carboxyfluorescein (FAM) channel (excitation = 487 nm, detection = 525 nm) for the acquisition of fluorescence data. Baseline threshold value of the amplification curve was calculated as 10 times standard deviation of the fluorescence signal during initial 5 min. Accordingly, for evaluation of the generation of “quasi” exponential amplification phase which is an indication of a positive reaction, each sigmoidal curve was analysed for a time-point corresponding to increase in florescence signal above threshold i.e. > 30 mV/min for a minimum of 3 consecutive measurements (Fig. 5). The time of first rise in signal above threshold (>30 mV/min) was given by the detection-time (Td) value. For unaided eye evaluation, 0.2 μl SYBR green fluorescent dye was added to the reaction tubes at the end of the assay. The positive reaction developed green colour while negative reaction remained orange. Furthermore, these reactions were analyzed for bright green fluorescence under UV light, which is an indication for a positive test. For agarose gel analysis, RT-LAMP products were incubated at 80 °C for 02 min to stop any residual enzymatic activity and subsequently analysed on 2% agarose gel stained with ethidium-bromide and the image was captured using a UV light transilluminator.

### Comparative evaluation of RT-LAMP assay with RT-PCR and/or Quantitative RT-PCR (RT-qPCR) using clinical samples

For comparative evaluation of LAMP with reverse transcription PCR (RT-PCR), PPRV nucleic acid was detected in the clinical samples that were collected from suspected goats in field outbreaks, after taking consent from their owners, and experimentally infected animals, bought from local market and kept under control conditions, from days 1–14 post-inoculation (dpi), by these tests. The progression of disease in these infected animals was monitored by measuring the cumulative clinical score and changes in rectal temperature. After 14 days, the animals were slaughtered and observed for any pathological lesions in the organs. RT-LAMP was applied directly on swab extracts as mentioned above. For RT-PCR and RT-qPCR based detection of PPRV in these samples, total RNA was extracted from 200 μl suspension of each swab-extract using TRIzol method following the manufacturer’s instructions (Invitrogen, Carlsbad, USA). RNA pellet was resuspended in 20 μl of RNase-free water, quantified using Nanodrop 1000 spectrophotometer, and stored at −80 °C until further use. Total RNA (1 μg) was reverse transcribed to cDNA by random hexamer primers using RevertAid™ First Strand cDNA Synthesis Kit following the manufacturer’s instructions (Invitrogen, Paisley, UK). Subsequently, 2.5 μl of first strand cDNA product was used as a template to amplify 463 bp fragment of nucleoprotein gene of PPRV by RT-PCR [33]. To test the sensitivity of the assay, serial dilutions of virus culture supernatants were subjected to the LAMP. For the comparative detection of PPRV genome, RNA extracted from tenfold serial dilutions ranging from 10^0^ to 10^−5^ dilution of PPRV starting from 10TCID_50_/ml was subjected to RT-LAMP and conventional RT-PCR recommended by OIE that amplifies a 351 bp fragment of the N gene of PPRV [1, 14]. One-step RT-qPCR was carried out, in parallel with the RT-LAMP assay, using the iScript One-Step RT-qPCR Kit (BioRad, Hercules, CA, USA). PPRV N-gene specific forward (5′- ggactgggcctcgacagg-3′) and reverse (5′-ggatcgcagctttgacttcttc-3′) primers were used in combination with Taqman probe (FAM-5′tccttcctccagcataa3′-BHQ1) [8]. For standard curve generation, seven 10-fold dilutions (10^6^-10^0^ copies) of a defined template (pTZ57R/T that contained partial CDS of PPRV N protein gene) were subjected to a 40-cycle qPCR assay along with clinical samples and no template control (NTC). The reaction was carried out in a total volume of 20 μl (400nM of each primer, 200nM of the probe and 0.4 μl of the iScript reverse transcriptase) using IQ5™ Real-Time PCR Detection System (BioRad, USA) under the following reaction conditions: 50 °C for 10 min; 95 °C for 5 min and 40 cycles of 95 °C for 10s and 60 °C for 30s.

## Additional files

Additional file 1: Detection limit of N-gene based RT-LAMP assay. Sheet 1; The excel file includes data as values of millivolts (mV) against specified time obtained from 10-fold Serial dilutions of fluid supernatant taken from cultured PPRV in CHS-20 cells harvested at appearance of 80% cytopathic effects. Sheet 2; The test was able to detect dilution up to 10^−4^ within 60 min. Sheet 3; includes data generated from real-time RT-PCR by amplifying 10-fold serial dilutions of known plasmid concentrations. Sheet 4; data generated in real-time RT-PCR based detection of PPRV in clinical samples collected in field outbreaks. (XLSX 51 kb)
Additional file 2: Data file generated by ESE software during the detection of PPRV in the field conditions. It is only readable format by software. (DAT 29 kb)
Additional file 3: Amplification curves obtained by ESE quant tube scanner software during the RT-LAMP based detection of PPRV in clinical samples. The slope of positive amplification curve becomes steeper during the exponential phase and becomes horizontal platue again as the reactions components are consumed during the amplification process. (PNG 153 kb)
Additional file 4: ESE Software file in notepad format generated from LAMP based PPRV detection in experimentally infected goat from 2-dpi to 12-dpi. In positive samples, the value of amplification signal in the form of mVolts increased with the passage of time until a platue phase is reached with no more increase in signal. (TXT 5 kb)
Additional file 5: RT-qPCR in goat. File includes information on amplification of PPRV from experimentally infected goat from 2 to 14-dpi. (RTF 3678 kb)
Additional file 6: ESE software file in notepad format generated from Detection of PPRV in clinical samples collected in outbreaks. Again in positive samples, the value of amplification signal in the form of mVolts increased with the passage of time during exponential phase until a platue phase is reached with no more increase in signal. (TXT 5 kb)
Additional file 7: RT-LAMP based detection of serial dilutions of virus supernatant. The graph was generated in “GraphPad Prism software” expressing increasing serial dilution of PPRV culture supernatant along x-axis and corresponding time of detection along y-axis. The RT-LAMP was able to detect up to 10^−4^TCID50/ml within 60 min. (TXT 143 bytes)
Additional file 8: Threshold validation in clinical samples. In the field outbreaks, only those samples were taken as positive which crossed the threshold limit of 30mVolts in their amplification signals. (PNG 164 kb)
Additional file 9: End-point UV visualization of RT-LAMP reaction. The positive samples produced bright green fluorescence in contrast to negative control. (JPG 981 kb)
Additional file 10: Data file of End point report of RT-qPCR. It includes the data of end-point analysis for threshold validation of amplification curves generated during the test. (RTF 261 kb)
Additional file 11: Data file of RT-qPCR based detection of serial dilutions of PPR N gene standard cloned plasmid, at FAM channel. Accuracy was equivalent to R^2^ = 0.999, 3.03 as value of slope. (RTF 3659 kb)
Additional file 12: Data file of RT-qPCR based detection of PPRV in clinical samples collected in field outbreaks. In these samples, ct values ranged from 22 to 33. (RTF 3690 kb)
Additional file 13: RT-PCR based detection of PPRV in blood of experimentally infected sheep and goat. Corresponding wells for Goat (G) and Sheep (S) are indicated in the figure along with respective number of day post inoculation (dpi). In goat virus detectable from 4-dpi to 14-dpi. (JPG 821 kb)

## Abbreviations

ASSURED: Affordable sensitive specific user-friendly robust equipment free deliverable to the end user
BIP: Backward inner primer
Bsm: *Bacillus smithii*
Bst: *Bacillus stearothermophilus*
cDNA: Complimentary DNA
cELISA: Competitive enzyme linked immunosorbent assay
CHS-20: Monkey kidney cells expressing sheep signaling lymphocyte activation molecule
dpi: Day post inoculation
FAM: 6- carboxyfluorescein
FAO: The Food and Agriculture Organization of the United Nations
FIP: Forward inner primer
FMDV: Foot and mouth disease virus
mV: Milli Volts
N: Nucleocapsid gene
NTCs: No template controls
OIE: The World Organization for Animal Health
PPR: Peste des petits ruminants
PPRV: Peste des petits ruminants virus
RT-LAMP: Reverse transcription-loop mediated isothermal amplification assay
RT-PCR: Reverse transcription polymerase chain reaction
TCID50/ml: Tissue culture infective dose-50 per ml
Td: Detection time
