# Supplementary figures and images for: Genetic detection of peste des petits ruminants virus under field conditions: a step forward towards disease eradication
Source: BMC Vet Res. 2017 Jan 25;13:34. doi: 10.1186/s12917-016-0940-0 (PMC5264299; doi:10.1186/s12917-016-0940-0)

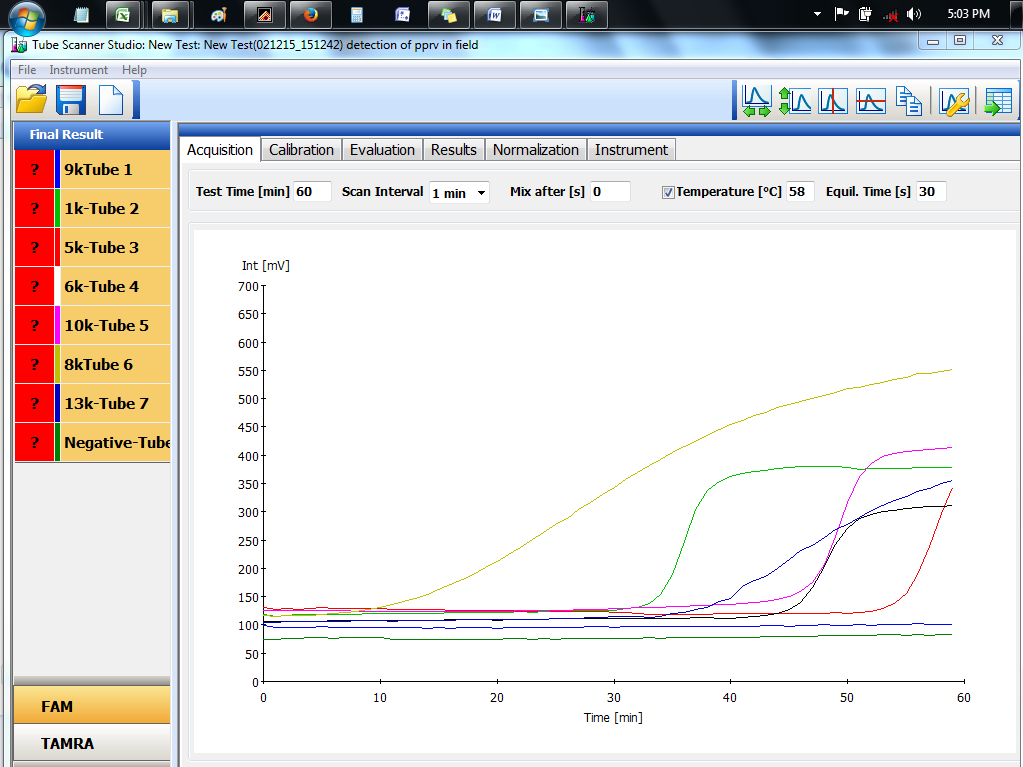

Supplement: Additional file 3: — Amplification curves obtained by ESE quant tube scanner software during the RT-LAMP based detection of PPRV in clinical samples. The slope of positive amplification curve becomes steeper during the exponential phase and becomes horizontal platue again as the reactions components are consumed during the amplification process. (PNG 153 kb) [file 12917_2016_940_MOESM3_ESM.png]

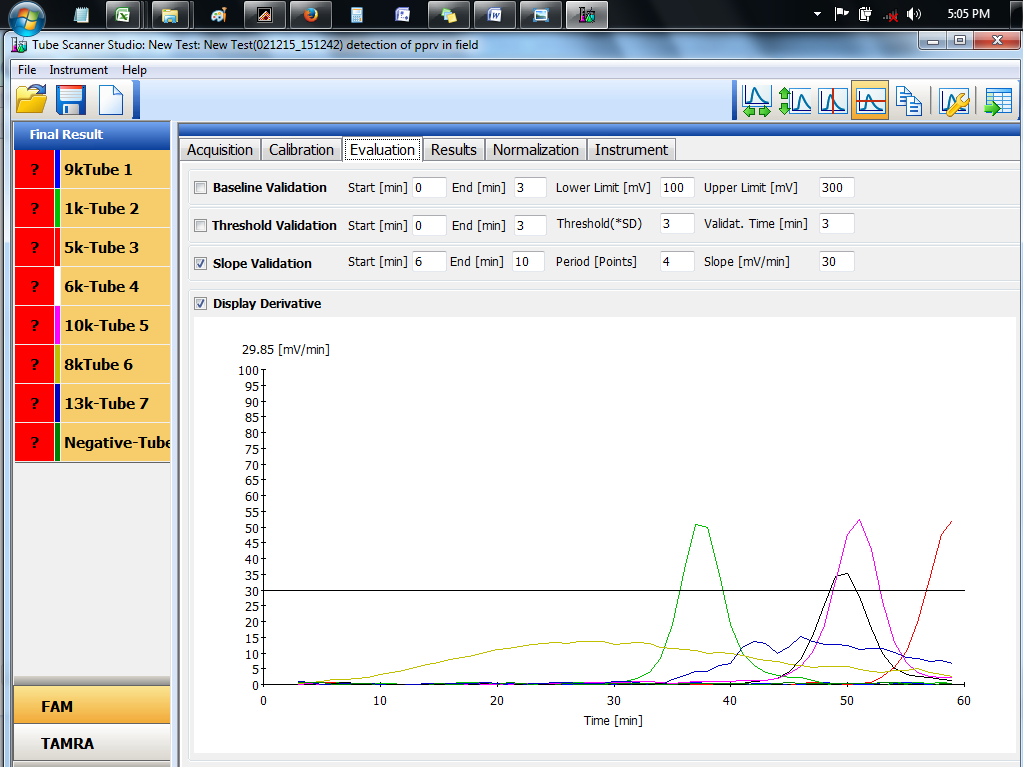

Supplement: Additional file 8: — Threshold validation in clinical samples. In the field outbreaks, only those samples were taken as positive which crossed the threshold limit of 30mVolts in their amplification signals. (PNG 164 kb) [file 12917_2016_940_MOESM8_ESM.png]

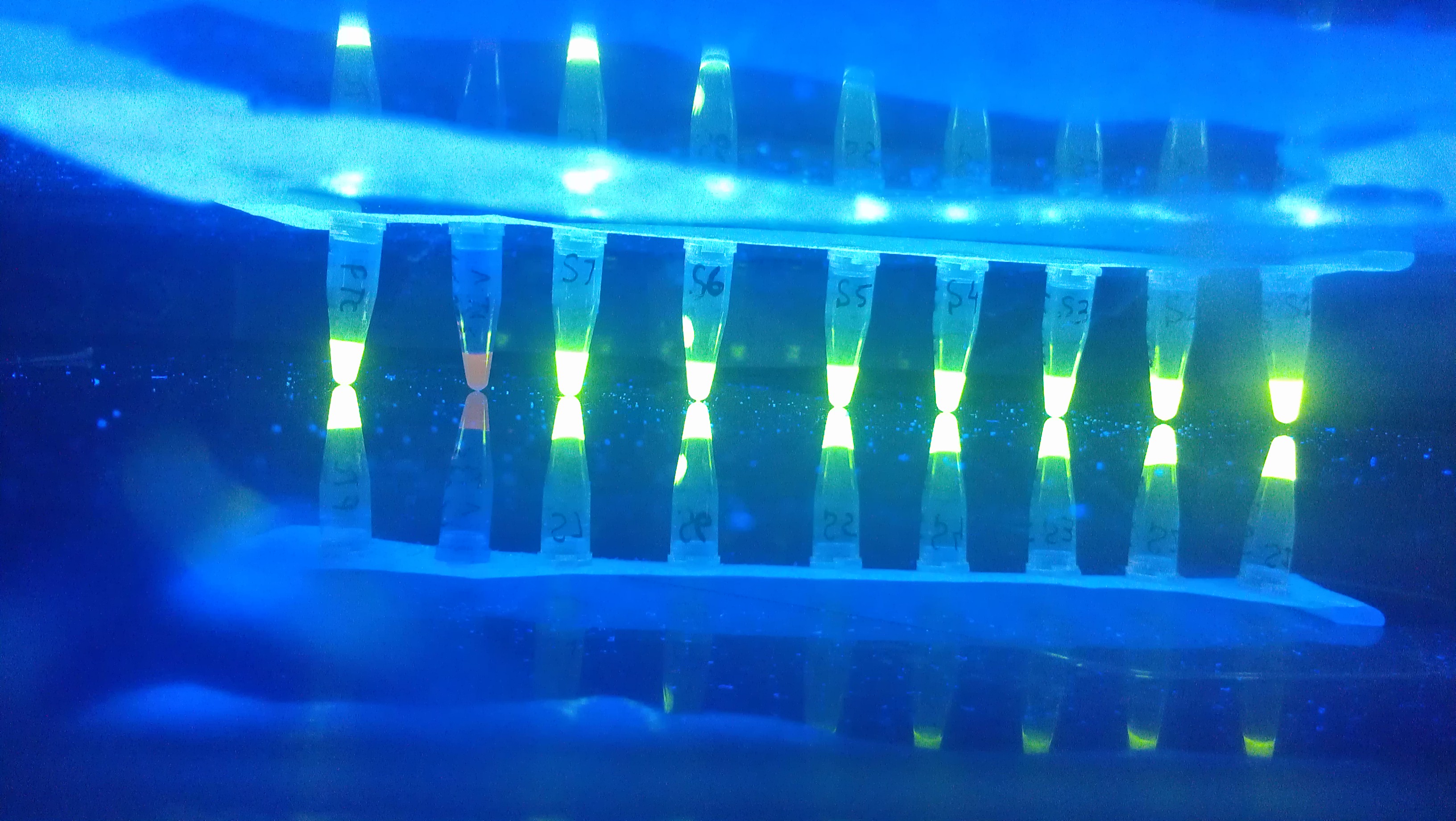

Supplement: Additional file 9: — End-point UV visualization of RT-LAMP reaction. The positive samples produced bright green fluorescence in contrast to negative control. (JPG 981 kb) [file 12917_2016_940_MOESM9_ESM.jpg]

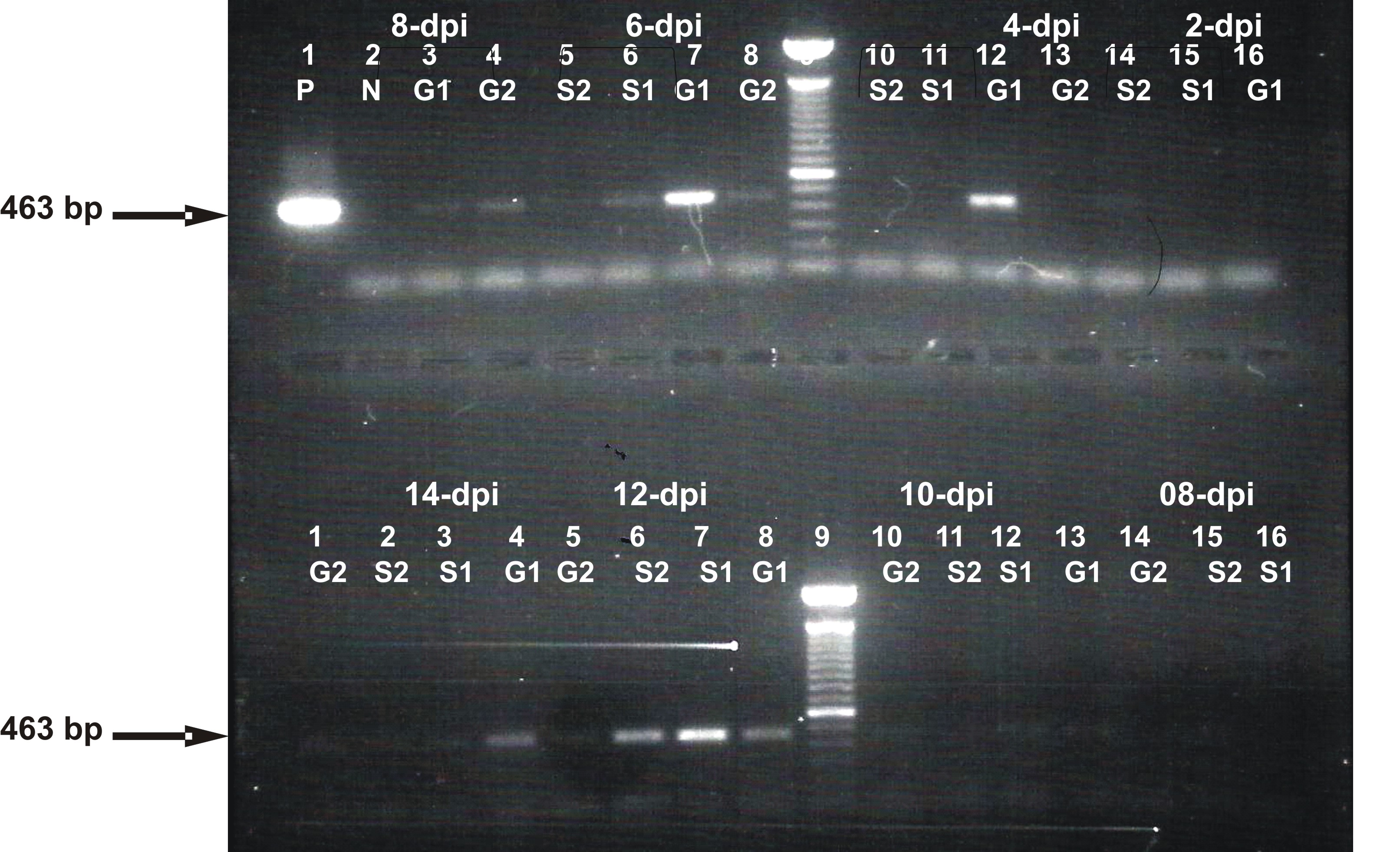

Supplement: Additional file 13: — RT-PCR based detection of PPRV in blood of experimentally infected sheep and goat. Corresponding wells for Goat (G) and Sheep (S) are indicated in the figure along with respective number of day post inoculation (dpi). In goat virus detectable from 4-dpi to 14-dpi. (JPG 821 kb) [file 12917_2016_940_MOESM13_ESM.jpg]
