# Supplementary material for: Genetic detection of peste des petits ruminants virus under field conditions: a step forward towards disease eradication
Source: BMC Vet Res. 2017 Jan 25;13:34. doi: 10.1186/s12917-016-0940-0 (PMC5264299; doi:10.1186/s12917-016-0940-0)
Supplement: Additional file 5: — RT-qPCR in goat. File includes information on amplification of PPRV from experimentally infected goat from 2 to 14-dpi. (RTF 3678 kb) [file 12917_2016_940_MOESM5_ESM.rtf]

PCR Quantification Detailed Report
PCR Base Line Subtracted Curve Fit Data (FAM)
Contains All Available Data 

General Data

OPD File Name:	waqas  PPR experiment 6-03-15.opd
OPD File Path:	C:\Program Files\Bio-Rad\iQ5\User1
Collected Data:	Collected Data
Current Date:	Friday, 06 March 2015
Run Date:	Friday, 06 March 2015
Active RMEs:	Original
Active Well Factors:	Persistent 
Background Readings Valid:	No, data is 100 day(s) old.
RME Valid:	Yes
Well Factors Valid:	No, data is 100 day(s) old.
Plate Setup File Name:	waqas  PPR experiment 6-03-15.pts
Plate Setup File Path:	C:\Program Files\Bio-Rad\iQ5\User1
Protocol File Name:	waqas PPRV experiment 06-03-2015.tmo
Protocol File Path:	C:\Program Files\Bio-Rad\iQ5\User1

Comments:    


Protocol:
	Cycle 1: (1X)
	Step 1:			50.0 °C			for 10:00.
	Step 2:			95.0 °C			for 05:00.
	Cycle 2: (40X)
	Step 1:			95.0 °C			for 00:15.
	Step 2:			60.0 °C			for 00:30.
	Data collection and real-time analysis enabled.
	Cycle 3: (1X)
	Step 1:			20.0 °C			for Hold.

PCR Quantification Data

PCR Amp/Cycle Chart


Standard Curve Data
	
Standard Curve Chart


	Fluor	PCR	R	Slope	y-Intercept
		Efficiency(%)	Squared		
	   FAM	 103.8	 0.974	-3.234	10.946

	Fluor	Units 	Quantity 	Original
		Changed?	Units	Units
	 FAM	  No	nanograms	nanograms

Number of valid standard wells:    None

Standard Curve Spreadsheet Data

	Fluor	Well	Type	Ident.	Rep	Ct	Log	SQ	SQ	SQ	Ct	Ct	Set
								SQ		Mean	SD	Mean	SD	Point
	FAM	D09	Std	-	7	29.65	-5.562	2.74E-06	2.74E-06	0.00E+00	29.85	0.275	N/A
	FAM	D08	Std	-	6	26.07	-4.562	2.74E-05	2.74E-05	0.00E+00	25.42	0.915	N/A
	FAM	D07	Std	-	5	21.33	-3.562	2.74E-04	2.74E-04	0.00E+00	21.32	0.002	N/A
	FAM	D04	Std	-	2	14.27	-0.562	2.74E-01	2.74E-01	0.00E+00	13.28	1.405	N/A
	FAM	C09	Unkn	-	8	34.29	-7.218	6.05E-08	5.12E-08	1.31E-08	34.55	0.363	N/A
	FAM	C08	Unkn	-	7	31.14	-6.245	5.69E-07	8.29E-07	3.67E-07	30.69	0.644	N/A
	FAM	C07	Unkn	-	6	33.47	-6.963	1.09E-07	1.38E-07	4.12E-08	33.17	0.425	N/A
	FAM	C06	Unkn	-	5	33.73	-7.044	9.04E-08	8.25E-08	1.11E-08	33.86	0.190	N/A
	FAM	C05	Unkn	-	4	34.97	-7.427	3.74E-08	5.12E-08	1.95E-08	34.58	0.550	N/A
	FAM	C04	Unkn	-	3	33.77	-7.056	8.79E-08	9.47E-08	9.61E-09	33.67	0.143	N/A
	FAM	C03	Unkn	-	2	35.37	-7.551	2.81E-08	2.82E-08	5.92E-11	35.37	0.003	N/A
	FAM	C02	Unkn	-	1	34.41	-7.255	5.56E-08	3.57E-08	2.81E-08	35.29	1.243	N/A
	FAM	B09	Unkn	-	8	34.81	-7.377	4.19E-08	5.12E-08	1.31E-08	34.55	0.363	N/A
	FAM	B08	Unkn	-	7	30.23	-5.963	1.09E-06	8.29E-07	3.67E-07	30.69	0.644	N/A
	FAM	B07	Unkn	-	6	32.87	-6.777	1.67E-07	1.38E-07	4.12E-08	33.17	0.425	N/A
	FAM	B06	Unkn	-	5	34.00	-7.127	7.46E-08	8.25E-08	1.11E-08	33.86	0.190	N/A
	FAM	B05	Unkn	-	4	34.19	-7.187	6.50E-08	5.12E-08	1.95E-08	34.58	0.550	N/A
	FAM	B04	Unkn	-	3	33.57	-6.993	1.02E-07	9.47E-08	9.61E-09	33.67	0.143	N/A
	FAM	B03	Unkn	-	2	35.36	-7.549	2.82E-08	2.82E-08	5.92E-11	35.37	0.003	N/A
	FAM	B02	Unkn	-	1	36.17	-7.799	1.59E-08	3.57E-08	2.81E-08	35.29	1.243	N/A
	FAM	E07	Std	-	5	21.32	-3.562	2.74E-04	2.74E-04	0.00E+00	21.32	0.002	N/A
	FAM	E09	Std	-	7	30.04	-5.562	2.74E-06	2.74E-06	0.00E+00	29.85	0.275	N/A
	FAM	E08	Std	-	6	24.77	-4.562	2.74E-05	2.74E-05	0.00E+00	25.42	0.915	N/A
	FAM	E04	Std	-	2	12.29	-0.562	2.74E-01	2.74E-01	0.00E+00	13.28	1.405	N/A
	FAM	A05	NTC	-	2	N/A	N/A	0.00E+00	0.00E+00	0.00E+00	.00	N/A	N/A
	FAM	A04	NTC	-	2	N/A	N/A	0.00E+00	0.00E+00	0.00E+00	.00	N/A	N/A
	FAM	A03	NTC	-	1	35.30	N/A	0.00E+00	0.00E+00	0.00E+00	35.60	0.425	N/A
	FAM	A02	NTC	-	1	35.90	N/A	0.00E+00	0.00E+00	0.00E+00	35.60	0.425	N/A


Run Parameters

	Hot Start?		No		
	Temperature Control Mode:    	Algorithmic     
	Volume:		25 ul

Data Analysis Parameters

Display Controls
	Fluor	Display Mode
	FAM	SinglePoint

Data Selection
	Fluor	Data Window 	Center
		Size
	FAM	99%	End

Digital Filtering
	Fluor	Global Filter	PCR Digital		Smoothing Filter 
		Enabled?	Filter Type		Desired Width
	FAM	Off	Weighted Mean		  5
	
PCR Data Analysis Method	
	Fluor	Data Analysis Method
	FAM	PCR Base Line Subtracted Curve Fit

PCR Baseline Data Analysis Parameters

Baseline Calculation
	Fluor	Baseline	Auto Baseline		Global Baseline Cycles
		 Method	Cycle Calculation?	Start	End
	  FAM	Data Window	  Yes		  N/A	  N/A

Overriden Baseline Cycles	None
				
				

Threshold Calculation
	Fluor	Use Auto	Auto Calculated	User Defined 
		Threshold?	Threshold Value	Threshold Value
	 FAM	  No	397.34		218.75

Excluded Wells
	Excluded Well Count:    	20

	Fluor	Well
	FAM	A6:<no identifier>
	FAM	A7:<no identifier>
	FAM	B10:<no identifier>
	FAM	B11:<no identifier>
	FAM	B12:<no identifier>
	FAM	C10:<no identifier>
	FAM	C11:<no identifier>
	FAM	C12:<no identifier>
	FAM	D3:<no identifier>
	FAM	D5:<no identifier>
	FAM	D6:<no identifier>
	FAM	D10:<no identifier>
	FAM	D11:<no identifier>
	FAM	D12:<no identifier>
	FAM	E3:<no identifier>
	FAM	E5:<no identifier>
	FAM	E6:<no identifier>
	FAM	E10:<no identifier>
	FAM	E11:<no identifier>
	FAM	E12:<no identifier>
 
Modified Wells
	Modified Well Count:	0

					
 			 	 	 
End
