# Supplementary material for: Genetic detection of peste des petits ruminants virus under field conditions: a step forward towards disease eradication
Source: BMC Vet Res. 2017 Jan 25;13:34. doi: 10.1186/s12917-016-0940-0 (PMC5264299; doi:10.1186/s12917-016-0940-0)
Supplement: Additional file 10: — Data file of End point report of RT-qPCR. It includes the data of end-point analysis for threshold validation of amplification curves generated during the test. (RTF 261 kb) [file 12917_2016_940_MOESM10_ESM.rtf]

End Point Analysis Detailed Report

General Data

OPD File Name:	waqas 01-01-2014.opd
OPD File Path:	C:\Program Files\Bio-Rad\iQ5\User1
Collected Data:	Collected Data
Current Date:	Tuesday, 01 January 2002
Run Date:	Wednesday, 01 January 2014
Active RMEs:	Original
Active Well Factors:	Persistent 
Background Readings Valid:	No, data is 41 day(s) old.
RME Valid:	Yes
Well Factors Valid:	No, data is 41 day(s) old.
Plate Setup File Name:	waqas 01-01-2014 DG Khan arifwala and balkasar.pts
Plate Setup File Path:	C:\Program Files\Bio-Rad\iQ5\User1
Protocol File Name:	waqas 01-01-2014.tmo
Protocol File Path:	C:\Program Files\Bio-Rad\iQ5\User1

Comments:    


Protocol:
	Cycle 1: (1X)
	Step 1:			50.0 °C			for 10:00.
	Step 2:			95.0 °C			for 05:00.
	Cycle 2: (35X)
	Step 1:			95.0 °C			for 00:15.
	Step 2:			60.0 °C			for 00:30.
	Data collection and real-time analysis enabled.
	Cycle 3: (1X)
	Step 1:			20.0 °C			for Hold.
 
Run Type: PCR Analysis

Fluorophore: FAM

End Point Analysis Spreadsheet Data

	Well	Sample	End	Define	Unknowns	Unknowns	Identifier
	Id	Type	RFUs	Controls	Call	Ranking
	A01	NTC	     32	(-) Negative		     	
	A02	NTC	      9	(-) Negative		     	
	A03	NTC	     24	(-) Negative		     	
	A04	NTC	     18	(-) Negative		     	
	A05	NTC	     23	(-) Negative		     	
	A06	NTC	     41	(-) Negative		     	
	A09	Unkn	     94			    1	
	A10	Unkn	     97			    1	
	A11	Unkn	    225			    1	
	A12	Unkn	    238			    1	
	B01	Unkn	     13			    1	
	B02	Unkn	     19			    1	
	B03	Unkn	     23			    1	
	B04	Unkn	     27			    1	
	B05	Unkn	     19			    1	
	B06	Unkn	     27			    1	
	B07	Unkn	    168			    1	
	B08	Unkn	    337		(+) Positive	    2	
	B09	Unkn	    297		(+) Positive	    2	
	B10	Unkn	    151			    1	
	B11	Unkn	     21			    1	
	B12	Unkn	     23			    1	
	C01	Unkn	     22			    1	
	C02	Unkn	     17			    1	
	C03	Unkn	     17			    1	
	C04	Unkn	     13			    1	
	C05	Unkn	    432		(+) Positive	    2	
	C06	Unkn	    195			    1	
	C07	Unkn	    250			    2	
	C08	Unkn	    230			    1	
	C09	Unkn	    348		(+) Positive	    2	
	C10	Unkn	    320		(+) Positive	    2	
	C11	Unkn	     85			    1	
	C12	Unkn	     48			    1	
	D01	Unkn	   1145		(+) Positive	    5	
	D02	Unkn	   1084		(+) Positive	    5	
	D03	Unkn	     55			    1	
	D04	Unkn	    148			    1	
	D05	Unkn	    677		(+) Positive	    3	
	D06	Unkn	    635		(+) Positive	    3	
	D07	Unkn	    440		(+) Positive	    2	
	D08	Unkn	    371		(+) Positive	    2	
	D09	Unkn	   1188		(+) Positive	    6	
	D10	Unkn	   1390		(+) Positive	    7	
	D11	Unkn	    280		(+) Positive	    2	
	D12	Unkn	    243			    2	
	E05	Unkn	    449		(+) Positive	    2	
	E06	Unkn	    517		(+) Positive	    3	
	E07	Unkn	    424		(+) Positive	    2	
	E08	Unkn	    420		(+) Positive	    2	
	E09	Unkn	    421		(+) Positive	    2	
	E10	Unkn	    484		(+) Positive	    3	
	E11	Unkn	    713		(+) Positive	    4	
	E12	Unkn	    713		(+) Positive	    4	
	F01	Unkn	     16			    1	
	F02	Unkn	     52			    1	
	F03	Unkn	    170			    1	
	F04	Unkn	    129			    1	
	F05	Unkn	     51			    1	
	F06	Unkn	     30			    1	
	F07	Unkn	     94			    1	
	F08	Unkn	    113			    1	
	F09	Unkn	     54			    1	
	F10	Unkn	     59			    1	
	F11	Unkn	     44			    1	
	F12	Unkn	     58			    1	
	G01	Unkn	     86			    1	
	G02	Unkn	    113			    1	
	G03	Unkn	     21			    1	
	G04	Unkn	     26			    1	
	G05	Unkn	     77			    1	
	G06	Unkn	     95			    1	
	G07	Unkn	     21			    1	
	G08	Unkn	     25			    1	
	G09	Unkn	    257		(+) Positive	    2	
	G10	Unkn	    260		(+) Positive	    2	
	G11	Unkn	    411		(+) Positive	    2	
	G12	Unkn	    421		(+) Positive	    2	
	H01	Std	   2190	(+) Positive		     	
	H02	Std	   2310	(+) Positive		     	
	H03	Std	   1832	(+) Positive		     	
	H04	Std	   1730	(+) Positive		     	
	H05	Std	   1271	(+) Positive		     	
	H06	Std	   1377	(+) Positive		     	

 
Unknowns falling within Defined Controls by outside of Tolerance


Well ID	End EFUs
H6		1377
H5		1271

Fluor	Units 	Quantity 	Original
		Changed?	Units	Units
	 FAM	  No	copy number	copy number

Run Parameters

	Hot Start?		No		
	Temperature Control Mode:    	Algorithmic     
	Volume:		25 ul

Data Analysis Parameters

Display Controls
	Fluor	Display Mode
	FAM	SinglePoint

Data Selection
	Fluor	Data Window 	Center
		Size
	FAM	99%	End

Digital Filtering
	Fluor	Global Filter	PCR Digital		Smoothing Filter 
		Enabled?	Filter Type		Desired Width
	FAM	Off	Weighted Mean		  5
	
PCR Data Analysis Method	
	Fluor	Data Analysis Method
	FAM	PCR Base Line Subtracted Curve Fit

End Point Analysis Parameters

	Number of Cycles	5
	Number of Ranks	10
	Lowest RFU value	9
	Highest RFU value	2310
	Current Method	Negatives
	Tolerance	RFUs
		230
	Range	2285
	+ Controls Avg RFU	1785
	+ Controls - Tolerance	1555
	- Controls Avg RFU	24
	- Controls + Tolerance	255

Excluded Wells
	Excluded Well Count:	6

	Fluor	Well
	FAM	H7:<no identifier>
	FAM	H8:<no identifier>
	FAM	H9:<no identifier>
	FAM	H10:<no identifier>
	FAM	H11:<no identifier>
	FAM	H12:<no identifier>
 
Modified Wells
	Modified Well Count:	0

					
 			 	 	 
End
