# Supplementary material for: Genetic detection of peste des petits ruminants virus under field conditions: a step forward towards disease eradication
Source: BMC Vet Res. 2017 Jan 25;13:34. doi: 10.1186/s12917-016-0940-0 (PMC5264299; doi:10.1186/s12917-016-0940-0)
Supplement: Additional file 11: — Data file of RT-qPCR based detection of serial dilutions of PPR N gene standard cloned plasmid, at FAM channel. Accuracy was equivalent to R2 = 0.999, 3.03 as value of slope. (RTF 3659 kb) [file 12917_2016_940_MOESM11_ESM.rtf]

PCR Quantification Detailed Report
PCR Base Line Subtracted Curve Fit Data (FAM)
Contains All Available Data 

General Data

OPD File Name:	waqas 10.12.13.opd
OPD File Path:	C:\Program Files\Bio-Rad\iQ5\User1
Collected Data:	Collected Data
Current Date:	Tuesday, 17 December 2013
Run Date:	Tuesday, 10 December 2013
Active RMEs:	Original
Active Well Factors:	Dynamic 
Background Readings Valid:	Yes
RME Valid:	Yes
Well Factors Valid:	Yes
Plate Setup File Name:	waqas  10.12.pts
Plate Setup File Path:	C:\Program Files\Bio-Rad\iQ5\User1
Protocol File Name:	waqas 10.12.13.tmo
Protocol File Path:	C:\Program Files\Bio-Rad\iQ5\User1

Comments:    


Protocol:
	Cycle 1: (1X)
	Step 1:			95.0 °C			for 00:30.
	Cycle 2: (2X)
	Step 1:			95.0 °C			for 01:00.
	Cycle 3: (1X)
	Step 1:			50.0 °C			for 02:00.
	Step 2:			95.0 °C			for 10:00.
	Cycle 4: (40X)
	Step 1:			95.0 °C			for 00:15.
	Step 2:			60.0 °C			for 01:00.
	Data collection and real-time analysis enabled.

PCR Quantification Data

PCR Amp/Cycle Chart


Standard Curve Data
	
Standard Curve Chart


	Fluor	PCR	R	Slope	y-Intercept
		Efficiency(%)	Squared		
	   FAM	 113.6	 0.999	-3.034	37.739

	Fluor	Units 	Quantity 	Original
		Changed?	Units	Units
	 FAM	  No	copy number	copy number

Number of valid standard wells:    None

Standard Curve Spreadsheet Data

	Fluor	Well	Type	Ident.	Rep	Ct	Log	SQ	SQ	SQ	Ct	Ct	Set
								SQ		Mean	SD	Mean	SD	Point
	FAM	F01	Std	-	6	25.57	4.000	1.00E+04	1.00E+04	0.00E+00	25.60	0.041	N/A
	FAM	D01	Std	-	4	19.56	6.000	1.00E+06	1.00E+06	0.00E+00	19.60	0.057	N/A
	FAM	F02	Std	-	6	25.63	4.000	1.00E+04	1.00E+04	0.00E+00	25.60	0.041	N/A
	FAM	D02	Std	-	4	19.64	6.000	1.00E+06	1.00E+06	0.00E+00	19.60	0.057	N/A
	FAM	B02	Std	-	2	13.57	8.000	1.00E+08	1.00E+08	0.00E+00	13.39	0.256	N/A
	FAM	B01	Std	-	2	13.21	8.000	1.00E+08	1.00E+08	0.00E+00	13.39	0.256	N/A
	FAM	G04	NTC	-	2	35.50	N/A	0.00E+00	0.00E+00	0.00E+00	35.10	0.568	N/A
	FAM	G03	NTC	-	2	34.70	N/A	0.00E+00	0.00E+00	0.00E+00	35.10	0.568	N/A
	FAM	G01	Std	-	7	28.79	3.000	1.00E+03	1.00E+03	0.00E+00	28.69	0.144	N/A
	FAM	E02	Std	-	5	22.38	5.000	1.00E+05	1.00E+05	0.00E+00	22.33	0.071	N/A
	FAM	E01	Std	-	5	22.28	5.000	1.00E+05	1.00E+05	0.00E+00	22.33	0.071	N/A
	FAM	G02	Std	-	7	28.59	3.000	1.00E+03	1.00E+03	0.00E+00	28.69	0.144	N/A
	FAM	C01	Std	-	3	16.92	7.000	1.00E+07	1.00E+07	0.00E+00	16.76	0.224	N/A
	FAM	C02	Std	-	3	16.60	7.000	1.00E+07	1.00E+07	0.00E+00	16.76	0.224	N/A
	FAM	A02	Std	-	1	10.50	9.000	1.00E+09	1.00E+09	0.00E+00	10.37	0.180	N/A
	FAM	A01	Std	-	1	10.24	9.000	1.00E+09	1.00E+09	0.00E+00	10.37	0.180	N/A


Run Parameters

	Hot Start?		No		
	Temperature Control Mode:    	Algorithmic     
	Volume:		25 ul

Data Analysis Parameters

Display Controls
	Fluor	Display Mode
	FAM	SinglePoint

Data Selection
	Fluor	Data Window 	Center
		Size
	FAM	99%	End

Digital Filtering
	Fluor	Global Filter	PCR Digital		Smoothing Filter 
		Enabled?	Filter Type		Desired Width
	FAM	Off	Weighted Mean		  5
	
PCR Data Analysis Method	
	Fluor	Data Analysis Method
	FAM	PCR Base Line Subtracted Curve Fit

PCR Baseline Data Analysis Parameters

Baseline Calculation
	Fluor	Baseline	Auto Baseline		Global Baseline Cycles
		 Method	Cycle Calculation?	Start	End
	  FAM	Data Window	  Yes		  N/A	  N/A

Overriden Baseline Cycles	None
				
				

Threshold Calculation
	Fluor	Use Auto	Auto Calculated	User Defined 
		Threshold?	Threshold Value	Threshold Value
	 FAM	  No	283.27		62.03

Excluded Wells
	Excluded Well Count:    	15

	Fluor	Well
	FAM	A3:<no identifier>
	FAM	A4:<no identifier>
	FAM	B3:<no identifier>
	FAM	B4:<no identifier>
	FAM	C3:<no identifier>
	FAM	C4:<no identifier>
	FAM	D3:<no identifier>
	FAM	D4:<no identifier>
	FAM	E3:<no identifier>
	FAM	E4:<no identifier>
	FAM	F3:<no identifier>
	FAM	F4:<no identifier>
	FAM	H1:<no identifier>
	FAM	H2:<no identifier>
	FAM	H3:<no identifier>
 
Modified Wells
	Modified Well Count:	0

					
 			 	 	 
End
