# Supplementary material for: Genetic detection of peste des petits ruminants virus under field conditions: a step forward towards disease eradication
Source: BMC Vet Res. 2017 Jan 25;13:34. doi: 10.1186/s12917-016-0940-0 (PMC5264299; doi:10.1186/s12917-016-0940-0)
Supplement: Additional file 12: — Data file of RT-qPCR based detection of PPRV in clinical samples collected in field outbreaks. In these samples, ct values ranged from 22 to 33. (RTF 3690 kb) [file 12917_2016_940_MOESM12_ESM.rtf]

PCR Quantification Detailed Report
PCR Base Line Subtracted Curve Fit Data (FAM)
Contains All Available Data 

General Data

OPD File Name:	waqas 23.12.13.opd
OPD File Path:	C:\Program Files\Bio-Rad\iQ5\User1
Collected Data:	Collected Data
Current Date:	Tuesday, 24 December 2002
Run Date:	Monday, 23 December 2013
Active RMEs:	Original
Active Well Factors:	Dynamic 
Background Readings Valid:	No, data is 32 day(s) old.
RME Valid:	Yes
Well Factors Valid:	Yes
Plate Setup File Name:	waqas 23.12.pts
Plate Setup File Path:	C:\Program Files\Bio-Rad\iQ5\User1
Protocol File Name:	waqasqRTPPRV18-11-13.tmo
Protocol File Path:	C:\Program Files\Bio-Rad\iQ5\User1

Comments:    


Protocol:
	Cycle 1: (1X)
	Step 1:			95.0 °C			for 00:30.
	Cycle 2: (2X)
	Step 1:			95.0 °C			for 01:00.
	Cycle 3: (1X)
	Step 1:			50.0 °C			for 10:00.
	Step 2:			95.0 °C			for 05:00.
	Cycle 4: (35X)
	Step 1:			95.0 °C			for 00:15.
	Step 2:			60.0 °C			for 00:30.
	Data collection and real-time analysis enabled.
	Cycle 5: (1X)
	Step 1:			20.0 °C			for Hold.

PCR Quantification Data

PCR Amp/Cycle Chart


Standard Curve Data
	
Standard Curve Chart


	Fluor	PCR	R	Slope	y-Intercept
		Efficiency(%)	Squared		
	   FAM	 105.0	 0.990	-3.208	41.733

	Fluor	Units 	Quantity 	Original
		Changed?	Units	Units
	 FAM	  No	copy number	copy number

Number of valid standard wells:    None

Standard Curve Spreadsheet Data

	Fluor	Well	Type	Ident.	Rep	Ct	Log	SQ	SQ	SQ	Ct	Ct	Set
								SQ		Mean	SD	Mean	SD	Point
	FAM	F08	Unkn	-	20	N/A	N/A	0.00E+00	0.00E+00	0.00E+00	.00	N/A	N/A
	FAM	F07	Unkn	-	20	N/A	N/A	0.00E+00	0.00E+00	0.00E+00	.00	N/A	N/A
	FAM	F06	Unkn	-	19	N/A	N/A	0.00E+00	0.00E+00	0.00E+00	.00	N/A	N/A
	FAM	F05	Unkn	-	19	N/A	N/A	0.00E+00	0.00E+00	0.00E+00	.00	N/A	N/A
	FAM	F04	Unkn	-	18	N/A	N/A	0.00E+00	0.00E+00	0.00E+00	.00	N/A	N/A
	FAM	F03	Unkn	-	18	N/A	N/A	0.00E+00	0.00E+00	0.00E+00	.00	N/A	N/A
	FAM	F02	Unkn	-	17	22.25	6.073	1.18E+06	1.17E+06	1.83E+04	22.27	0.022	N/A
	FAM	F01	Unkn	-	17	22.29	6.063	1.16E+06	1.17E+06	1.83E+04	22.27	0.022	N/A
	FAM	E12	Unkn	-	16	N/A	N/A	0.00E+00	0.00E+00	0.00E+00	.00	N/A	N/A
	FAM	E11	Unkn	-	16	N/A	N/A	0.00E+00	0.00E+00	0.00E+00	.00	N/A	N/A
	FAM	E10	Unkn	-	15	29.09	3.942	8.74E+03	1.66E+04	1.11E+04	28.37	1.014	N/A
	FAM	E09	Unkn	-	15	27.66	4.389	2.45E+04	1.66E+04	1.11E+04	28.37	1.014	N/A
	FAM	E08	Unkn	-	14	32.64	2.836	6.86E+02	9.38E+02	3.56E+02	32.25	0.542	N/A
	FAM	E07	Unkn	-	14	31.87	3.075	1.19E+03	9.38E+02	3.56E+02	32.25	0.542	N/A
	FAM	E06	Unkn	-	13	29.92	3.683	4.81E+03	5.84E+03	1.45E+03	29.67	0.350	N/A
	FAM	E05	Unkn	-	13	29.43	3.837	6.87E+03	5.84E+03	1.45E+03	29.67	0.350	N/A
	FAM	E04	Unkn	-	12	26.51	4.746	5.57E+04	5.43E+04	1.98E+03	26.55	0.051	N/A
	FAM	E03	Unkn	-	12	26.58	4.723	5.29E+04	5.43E+04	1.98E+03	26.55	0.051	N/A
	FAM	E02	NTC	-	2	N/A	N/A	0.00E+00	0.00E+00	0.00E+00	.00	N/A	N/A
	FAM	E01	NTC	-	2	N/A	N/A	0.00E+00	0.00E+00	0.00E+00	.00	N/A	N/A
	FAM	D08	Std	-	6	28.93	4.000	1.00E+04	1.00E+04	0.00E+00	28.90	0.037	N/A
	FAM	D07	Std	-	6	28.87	4.000	1.00E+04	1.00E+04	0.00E+00	28.90	0.037	N/A
	FAM	D06	Std	-	5	25.73	5.000	1.00E+05	1.00E+05	0.00E+00	25.34	0.544	N/A
	FAM	D05	Std	-	5	24.96	5.000	1.00E+05	1.00E+05	0.00E+00	25.34	0.544	N/A
	FAM	D04	Std	-	4	22.74	6.000	1.00E+06	1.00E+06	0.00E+00	23.37	0.884	N/A
	FAM	D03	Std	-	4	23.99	6.000	1.00E+06	1.00E+06	0.00E+00	23.37	0.884	N/A
	FAM	D02	Std	-	3	18.79	7.000	1.00E+07	1.00E+07	0.00E+00	18.81	0.017	N/A
	FAM	D01	Std	-	3	18.82	7.000	1.00E+07	1.00E+07	0.00E+00	18.81	0.017	N/A
	FAM	C12	Std	-	2	16.10	8.000	1.00E+08	1.00E+08	0.00E+00	15.81	0.411	N/A
	FAM	C11	Std	-	2	15.52	8.000	1.00E+08	1.00E+08	0.00E+00	15.81	0.411	N/A
	FAM	C10	Std	-	1	13.07	9.000	1.00E+09	1.00E+09	0.00E+00	13.07	0.000	N/A
	FAM	C09	Std	-	1	13.08	9.000	1.00E+09	1.00E+09	0.00E+00	13.07	0.000	N/A
	FAM	C08	Unkn	-	10	31.79	3.100	1.26E+03	1.97E+03	1.00E+03	31.26	0.742	N/A
	FAM	C07	Unkn	-	10	30.74	3.427	2.67E+03	1.97E+03	1.00E+03	31.26	0.742	N/A
	FAM	C06	Unkn	-	9	N/A	N/A	0.00E+00	0.00E+00	0.00E+00	.00	N/A	N/A
	FAM	C05	Unkn	-	9	N/A	N/A	0.00E+00	0.00E+00	0.00E+00	.00	N/A	N/A
	FAM	C04	Unkn	-	8	30.82	3.404	2.53E+03	2.93E+03	5.58E+02	30.63	0.267	N/A
	FAM	C03	Unkn	-	8	30.44	3.521	3.32E+03	2.93E+03	5.58E+02	30.63	0.267	N/A
	FAM	C02	Unkn	-	7	N/A	N/A	0.00E+00	0.00E+00	0.00E+00	.00	N/A	N/A
	FAM	C01	Unkn	-	7	33.29	2.633	4.30E+02	4.30E+02	0.00E+00	33.29	N/A	N/A
	FAM	B12	Unkn	-	6	31.73	3.117	1.31E+03	1.76E+03	6.38E+02	31.37	0.516	N/A
	FAM	B11	Unkn	-	6	31.00	3.345	2.21E+03	1.76E+03	6.38E+02	31.37	0.516	N/A
	FAM	B10	Unkn	-	5	31.39	3.224	1.68E+03	2.00E+03	4.60E+02	31.16	0.323	N/A
	FAM	B09	Unkn	-	5	30.93	3.367	2.33E+03	2.00E+03	4.60E+02	31.16	0.323	N/A
	FAM	B08	Unkn	-	4	N/A	N/A	0.00E+00	0.00E+00	0.00E+00	.00	N/A	N/A
	FAM	B07	Unkn	-	4	32.29	2.945	8.81E+02	8.81E+02	0.00E+00	32.29	N/A	N/A
	FAM	B06	Unkn	-	3	32.35	2.925	8.41E+02	1.06E+03	3.16E+02	32.05	0.420	N/A
	FAM	B05	Unkn	-	3	31.76	3.110	1.29E+03	1.06E+03	3.16E+02	32.05	0.420	N/A
	FAM	B04	Unkn	-	2	30.76	3.422	2.64E+03	2.64E+03	0.00E+00	30.76	N/A	N/A
	FAM	B03	Unkn	-	2	N/A	N/A	0.00E+00	0.00E+00	0.00E+00	.00	N/A	N/A
	FAM	B02	Unkn	-	1	N/A	N/A	0.00E+00	0.00E+00	0.00E+00	.00	N/A	N/A
	FAM	B01	Unkn	-	1	N/A	N/A	0.00E+00	0.00E+00	0.00E+00	.00	N/A	N/A
	FAM	A04	NTC	-	1	N/A	N/A	0.00E+00	0.00E+00	0.00E+00	.00	N/A	N/A
	FAM	A03	NTC	-	1	N/A	N/A	0.00E+00	0.00E+00	0.00E+00	.00	N/A	N/A
	FAM	A02	NTC	-	1	N/A	N/A	0.00E+00	0.00E+00	0.00E+00	.00	N/A	N/A
	FAM	A01	NTC	-	1	N/A	N/A	0.00E+00	0.00E+00	0.00E+00	.00	N/A	N/A


Run Parameters

	Hot Start?		No		
	Temperature Control Mode:    	Algorithmic     
	Volume:		25 ul

Data Analysis Parameters

Display Controls
	Fluor	Display Mode
	FAM	SinglePoint

Data Selection
	Fluor	Data Window 	Center
		Size
	FAM	99%	End

Digital Filtering
	Fluor	Global Filter	PCR Digital		Smoothing Filter 
		Enabled?	Filter Type		Desired Width
	FAM	Off	Weighted Mean		  5
	
PCR Data Analysis Method	
	Fluor	Data Analysis Method
	FAM	PCR Base Line Subtracted Curve Fit

PCR Baseline Data Analysis Parameters

Baseline Calculation
	Fluor	Baseline	Auto Baseline		Global Baseline Cycles
		 Method	Cycle Calculation?	Start	End
	  FAM	Data Window	  Yes		  N/A	  N/A

Overriden Baseline Cycles	None
				
				

Threshold Calculation
	Fluor	Use Auto	Auto Calculated	User Defined 
		Threshold?	Threshold Value	Threshold Value
	 FAM	 Yes	113.53		113.53

Excluded Wells
	Excluded Well Count:    	0

		
		
 
Modified Wells
	Modified Well Count:	0

					
 			 	 	 
End
